# Digital Twin for the Production of hMSC-Derived Extracellular Vesicles for Applications in Cell and Gene Therapy toward Autonomous Operation

**DOI:** 10.1021/acsomega.5c03703

**Published:** 2025-09-30

**Authors:** Alina Hengelbrock, Simon Baukmann, Alexander Uhl, Axel Schmidt, Jochen Strube

**Affiliations:** Institute for Separation and Process Technology, 26534 Clausthal University of Technology, Clausthal-Zellerfeld 38678, Germany

## Abstract

Cell and gene therapy are innovative advanced therapy medicinal products (ATMPs) whose importance for the treatment of various rare and challenging diseases has increased significantly in recent years. Due to their versatility and more flexible application, extracellular vesicles (EVs) show great potential compared to classical cell therapy. Autonomous processes help to reduce the facility footprint and the cost of goods (COG). This requires calibrated predictive process models which, in combination with state-of-the-art process analytical technologies (PAT) and an advanced process control (APC) strategy, guarantee consistent product quality in line with the quality-by-design (QbD) approach required by the regulatory authorities. In this study, a process was therefore developed that consists of two chromatographic purification steps after 3D cultivation of human mesenchymal stem cells (hMSC) on microcarriers and harvesting by depth filtration. The optimized multimodal size exclusion chromatography (SEC) is used for the efficient removal of proteins, and in the subsequent anion exchange chromatography (AEX), the strengths in DNA removal as well as the separation of intact and nonintact EVs are exploited. With a clearance of >98% of DNA and proteins, the regulatory thresholds with regard to DNA per dose and protein concentration are met. The required physicochemical-based mechanistic process models were calibrated for all unit operations. Furthermore, the potential of *in situ* microscope imaging to determine the viable cell density for adherent growing hMSC during the upstream processing (USP) stage is demonstrated. Fourier transform infrared (FTIR) spectroscopy is used to predict the concentration of the critical metabolite glucose. Inline multi-angle light scattering (MALS) is established in the downstream stage to determine the total particle concentration. The developed predictive process models and PAT tools resulted in the advanced process control (APC) strategy and pave the way for a fully automated process that enables additional productivity increases of 20% at 99.9% reliability with a time saving factor of 2. In summary, this study helps to further improve process economics by reducing batch failures, time-to-market, and COG, which makes these innovations accessible to more patients. Robust, scalable processes are required for commercial production.

## Graphical abstract

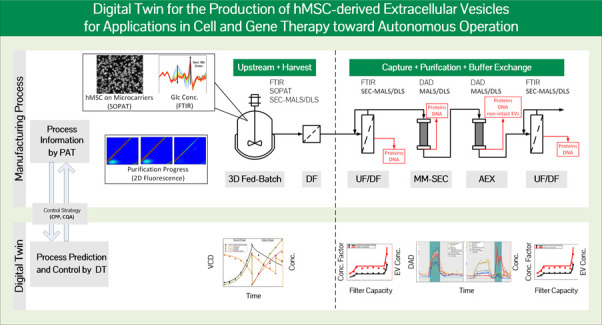

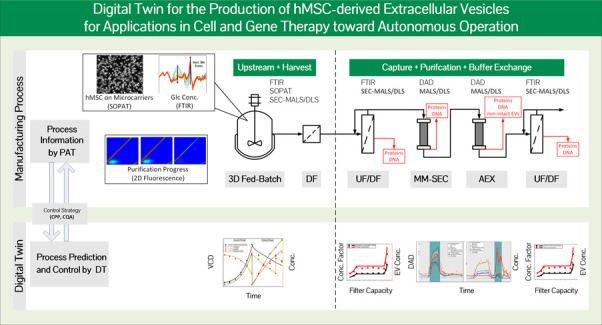

## 1 Introduction

Extracellular vesicles (EVs) are nanoscale vesicles that are an important part of the intercellular communication in the human body and are considered a promising therapeutic active substance in cell and gene therapy.^1–9^ A milestone for the therapeutic EV field has been the recent FDA approval of Ryoncil, an allogeneic bone marrow-derived mesenchymal stromal cell therapy.^10^ Communication takes place through the transfer of proteins, lipids, ribonucleic acids (RNA), and metabolites.^11^ Depending on their size and secretion mechanism, EVs can be divided into different groups: apoptotic bodies (50–1000 nm), microvesicles (100–350 nm), and exosomes (30–150 nm).^4,11–15^

EVs are secreted by all cells into the extracellular space and mediate both physiological and pathological processes by influencing the cellular microenvironment.^3,4,13,16–21^ Due to the specificity of their cargos and the easy accessibility in almost all physiological fluids and solid tissues,^14,22,23^ EVs are promising biomarkers in the early diagnosis of various diseases.^5–8,23^ Furthermore, as natural carriers of cargos such as proteins and RNA, they are well suited for drug delivery.^7,8,20,21,24,25^ Drug loading can be achieved by various methods, e.g., during cultivation by adding additives to the cell culture medium, genetic modification of the production cell line or after cultivation by ultrasonic treatment.^26–29^

Mesenchymal stem cells (MSC) in particular have proven to be effective as a production organism in cell therapy.^1,30,31^ MSC are multipotent cells that are found in many adult tissues.^32,33^ They play a central role in tissue repair and regeneration as well as immune modulation^31^ and contribute to the maintenance of homeostasis.^32,33^ In addition, (human) MSC (hMSC) are easily isolated and highly proliferated *in vitro*,^31^ exhibiting strong paracrine secretion of EVs.^7,34–36^

An essential requirement for the use of EVs instead of classic cell therapy using hMSC is the preservation of the therapeutic effect of the parent cells.^36–39^ In this way, the safety and availability of the therapy can be increased.^2,40^ In addition, the possibility of using EVs as vehicles for the protected transport of drugs across biological barriers^41^ opens up new versatile, flexible, and cost-effective^42^ possibilities for developing rapidly available therapies.

## 2 Fundamentals and State-of-the-Art

The robust and reliable production of EVs requires a scalable, reproducible process that delivers consistent product quality.^1,3,43^

The growth of hMSC is adherent by default, although research is moving toward the implementation of suspension cultures.^44^ The adherent growth of hMSC has caused challenges for scale-up. The most common method is to maximize the growth surface, using mainly hollow fiber bioreactors or microcarriers.^45–48^ The latter are mostly used either in a vertical wheel reactor or in a stirred bioreactor.^27,49–53^ The biggest challenge is to maintain the phenotype during the transition from planar 2D systems such as T-flasks to well-mixed 3D environments.^43^ Due to air bubbles during aeration through microsparger and agitation, shear stress is introduced to the cells, which could lead to osteogenic differentiation.^54,55^ Furthermore, shear stress should be avoided as much as possible in order to minimize cell death and the formation of apoptotic bodies.^56,57^ To maximize the yield, longer process times are aimed at, whereby a substrate limitation must be avoided. Traditionally, this was achieved by multiple media exchanges,^58–61^ but in recent years, this has been reduced to minimized volumes of feed,^62–64^ and if necessary, a simple media exchange.^52,65–68^ This was primarily necessary due to the high (nonproduct) number of particles in the medium, which would pose challenges in analytics and downstream, so that a low-particulate medium is used to collect the particles.^52,67,68^ Recent studies show further progress toward a process without media exchange, whereby the overall process duration remained comparable, but a continuous EV production could be achieved, resulting in at least twice the yield compared to the reference process.^69^

At the end of cultivation, various impurities are present in the medium, which must be removed by using suitable purification methods. In addition to nonconsumed metabolites, there are regulatory concerns and requirements for genomic, double-stranded DNA (dsDNA) and host cell proteins (HCP) in particular.^1^ Additionally, there are impurities in the size range of the target EVs such as apoptotic vesicles or product aggregates.

A widely used method, especially in laboratory processes, is ultracentrifugation (UC), which enables purification based on size differences.^7,13,70–73^ However, this method does not achieve as high purities and yields as other size-based separation methods.^74–79^ Further challenges are the partial destruction of the vesicles and an increased aggregation rate.^80–82^ In addition, it is often time-consuming and cost-intensive, and due to the high rotational speeds required, only limited scaling is possible.^8,12,26,43,83^ Alternatively, polymer-based precipitation has established itself as a cost-effective and scalable method, also in commercially available kits.^1,31^ Due to the coprecipitation of impurities, low purity is also the result here.^84^

Size-based separation methods like tangential flow filtration (TFF) are also widely used in the purification of EVs.^13,79,85,86^ This enables significantly higher yields and purities compared to UC.^74^ Due to their similar structure to enveloped viruses^87^ and their derived virus-like particles (VLPs), special attention should be paid to the selection of suitable process conditions to prevent disruption of the shear-sensitive particles.^88,89^ With regard to subsequent chromatography steps or stabilization, buffer exchange including concentration adjustment is also possible.^90^

Purification by size exclusion chromatography (SEC) also leads to higher purity compared to UC, but also to co-isolation of other cellular products.^74,91^ Another disadvantage of SEC is the high dilution^92–94^ and low loading capacity,^93,94^ which is why it is mostly used as a final purification step in bioprocesses. As an alternative, multimodal SEC columns^3,81,94^ have been developed whose beads are internally functionalized. Particles larger than the exclusion limit are thus separated by their respective sizes, while smaller particles can diffuse into the beads and are bound.

Separations based on size enable the removal of impurities such as proteins, but particles of a comparable size to the product cannot be separated.^43^ Affinity methods are proposed to separate these as well.^7,13,43,72,95^ In addition to high consumption costs,^7,13^ a corresponding ligand that binds a specific target molecule is required. These can be surface markers such as the tetraspanins^43^ characteristic of EVs or surface cargos. Besides high costs,^12,96^ the latter would limit the flexibility of a process if EVs are to be purified with different cargos. The amount of expressed tetraspanins, on the other hand, can vary with each donor and from batch to batch, resulting in fluctuations in purification that would have to be compensated for by subsequent process steps.^97–101^

One chromatography method that is already established for other extracellular vesicles such as adenoviruses or virus-like particles is intermediate purification using anion exchange chromatography (AEX).^12,102–106^ It enables a more cost-effective and scalable separation due to the negative charge of the EVs. In addition to the removal of proteins and DNA, the separation of intact, tetraspanin-positive and nonintact, tetraspanin-negative EVs is also possible.^107^

The need for automated processes has already been reported in the literature and first concepts toward automation for traditional processes, consisting of cultivation followed by TFF or ultracentrifugation, have been presented.^108^ As discussed above, these processes offer significant disadvantages in terms of their scalability and purity. The concepts focus on automation of the individual process steps. Concepts for automating the overall process, as already demonstrated for other material systems,^103,109,110^ can significantly reduce the equipment footprint, the cost of goods (COG), and avoid hold times.^111^

Key enablers are digital twins (DT) and appropriate process analytical technologies (PAT), which are supported by quality-by-design (QbD) principles.^112–114^ QbD-based processes are required by regulatory authorities and are becoming the standard in the biopharmaceutical industry.^115–117^ A control strategy is essential to achieve the desired quality target product profile (QTPP). With the help of validated DTs, design spaces (DS) can be created that enable the development and application of advanced process control (APC). This enables the operating point to remain within a defined range around the optimum operating point^118^ in the DS. In addition to reducing operating costs and production costs, the risk of out-of-specification (OOS) runs and batch failures can be minimized. In addition, there are significant savings in quality assurance (QA) by enabling real-time release testing (RTRT).^119^ The latter are based on the use of PAT, which are also essential for the automation of the process, as they measure process data online in real time and forward it to the digital twin. In addition to classic PAT such as conductivity, pH value, and pressure, spectroscopic methods such as Fourier transform infrared (FTIR), Raman, and UV–vis are becoming increasingly important.^102,110,120,121^ They enable the analysis of critical process variables such as product and impurity concentrations which is essential for the use of DT as well as RTRT.^122–124^

This study should help to further improve process economics by reducing batch failures, time-to-market, and COG, which makes these innovations accessible to more patients. Robust, scalable processes are required for commercial production. By demonstrating the adaptability of process control strategies of similar modalities like lentivirus-like particles, we also highlight the unique differences, like the adherent growth of hMSC versus other producer cell systems. QbD and PAT as a framework and guidance for others to start their process development.

The aim of this study is, therefore, to develop a robust and scalable production process for the production of EVs (see Figure 1). After the 3D cultivation of hMSC, EVs were harvested using depth filtration. Subsequently, an initial purification and buffer exchange via ultrafiltration and diafiltration (UFDF) was carried out, followed by a multimodal SEC. The product pool was loaded onto an AEX. Parallel to the process development, the required model parameters for the DT were determined and possible PAT were investigated. These are the key components for the control strategy with regard to an automated process, controlled with the help of APC.

**Figure 1:**
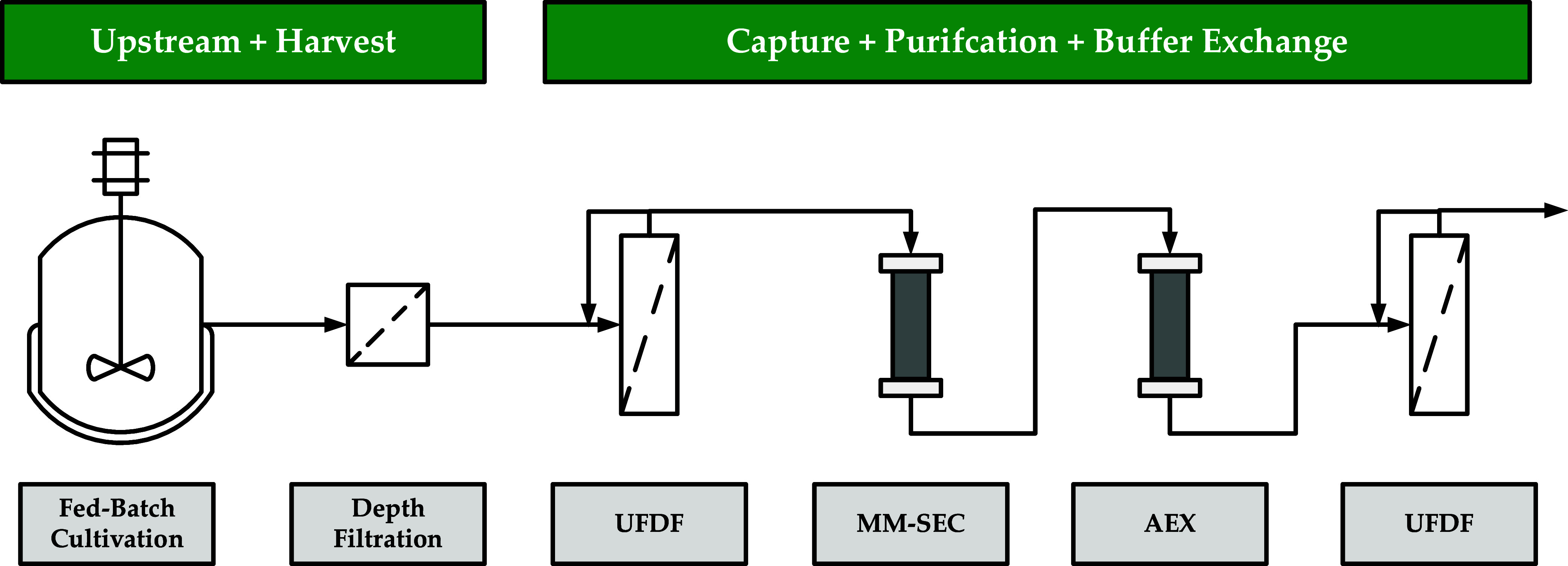
Overview of the EV production process.

## 3 Materials and Methods

### 3.1 Cultivation of Human Mesenchymal Stem Cells

The hMSCs were cultivated according to the manufacturer’s instructions (BM hMSC, Rooster Bio Inc., Frederick, MD, USA) in a 2 L glass bioreactor (Sartorius, Göttingen, Germany) on microcarriers (Solohill Microcarriers, Sartorius, Göttingen, Germany). After 3 days of growth phase in RoosterNourish XF, the cultivation was fed with RoosterReplenisch, and after 5 days, a medium exchange to EV Collect Media (all three: Rooster Bio Inc., Frederick, MD, USA) was performed. The collection phase lasted for another 5 days.

### 3.2 Harvest by Depth Filtration

Free cells and cell debris were removed by depth filtration with Milistak+ D0HC (cutoff: 0.55–9 μm, Merck KGaA, Darmstadt, Germany). The filtration setup consisted of a shear-reduced membrane pump (QuattroFlow, Quattroflow Fluid Systems GmbH & Co. KG, Hardegsen, Germany) and pressure sensors up- and downstream of the filter.

### 3.3 Ultra- and Diafiltration

The initial purification and concentration of the product was carried out using a hollow fiber module with a cutoff of 750 kDa (Spectrum MidiKros D02-E750-05-N, Repligen Corporation, Waltham, MA, USA). A shear rate of 4000 s^–1^ and a transmembrane pressure of 0.33 bar were applied.^125^ After concentration by a factor of 2.2, a buffer exchange with 5 diafiltration volumes was carried out on the Capto Core 400 running buffer. Finally, a second concentration (factor of 2.2) was performed.

### 3.4 Chromatography

#### 3.4.1 Multimodal SEC

The retentate of the UFDF was further purified with the multimodal Capto Core 400 (Cytiva, Marlborough, MA, USA). The shell of the beads is unfunctionalized, and the core is functionalized (AEX and HIC). After 5 CV equilibration (3 mL/min), 1 CV of the clarified harvest was loaded onto the column^126^ and then washed for 10 CV. This was done at 0.8 mL/min, and the buffer was 50 mM HEPES with 154 mM NaCl at pH 7.5. CIP was performed according to the manufacturer’s instructions in reversed flow at 0.5 mL/min with 1 M NaOH and 30% isopropanol. The UV absorbance was recorded at 280 nm and additionally at 260 nm. In addition, the particle size and number were determined using MALS/DLS postcolumn.

#### 3.4.2 Anion Exchange Chromatography

Furthermore, a strong anion exchanger (1 mL, Poros HQ, Thermo Fisher Scientific Inc., Waltham, MA, USA) was investigated. The mobile phase A (MPA) consisted of 50 mM HEPES with 180.7 mM NaCl at pH 7.5, and the elution buffer (MPB) contained 50 mM HEPES with 1 M NaCl at pH 7.5.^1^

After equilibration of 4 CV with MPA, 10 CV of the UFDF retentate was loaded onto the column. Since the UFDF retentate has a lower salt concentration than the MPA, an additional 3.1% MPB was added during the load. This was followed by washing for 4 CV with MPA. Subsequently, elution was carried out through 20 CV with a linear gradient from 0 to 100% MPB. After re-equilibration with 4 CV of MPA, a CIP step was applied for 10 CV with 1 M NaOH and 2 M NaCl. The flow rate was set to 1 mL/min, and fractions with a volume of 1.5 mL were collected.^1^ The UV absorbance was recorded at 280 nm and additionally at 260 nm. In addition, the particle size and number were determined by using MALS/DLS behind the column.

### 3.5 Analytical Methods

#### 3.5.1 PicoGreen dsDNA Detection

The DNA concentration was determined using the Quant iTTM PicoGreen dsDNA Reagent Kit (Thermo Fisher Scientific Inc., Waltham, USA) and performed according to the manufacturer’s instructions for 96-microtiter-well plates. Due to the low concentrations during chromatography, in addition to the standard calibration curve (2.5–1000 ng/mL), one was prepared for the low concentration range (2.5–150 ng/mL). The excitation wavelength was set to 480 nm, and the emission of the samples measured in duplicates was determined at 520 nm.

#### 3.5.2 Bradford and BCA Assay

The total protein concentration was analyzed with the Pierce Bradford Protein Assay Kit (Thermo Fisher Scientific Inc., Waltham, USA) using the microwell method according to the manufacturer’s instructions. Since some substances such as glucose or sodium chloride can interfere with the Bradford assay, the BCA assay kit (Thermo Fisher Scientific Inc., Waltham, USA) was additionally used for the chromatography samples.

#### 3.5.3 Nanoparticle Tracking Analysis

The particle concentration was determined using nanoparticle tracking analysis (NTA) at a wavelength of 520 nm with the ZetaView x30 (Particle Metrix GmbH, Ammersee, Germany). In order to maintain a measurement range of 1 × 10^7^–1 × 10^8^ particles/mL, the samples were diluted accordingly with deionized water. Three measurements were performed each with a total image acquisition time of 60 s over 5 cycles.

#### 3.5.4 MALS/DLS

In addition to the NTA measurements, a MALS/DLS detector (DAWN, Wyatt Technology, Santa Barbara, CA, USA) was used for particle analysis downstream of the chromatography. Particle size and number were analyzed by using ASTRA 8.1.2 software. The light scattering data was analyzed using the spherical model, assuming that the particles are spherical, have a shell thickness of 10 nm, and a refractive index of *n* = 1.41.^127–129^ The hydrodynamic radius was calculated from the DLS signal.

#### 3.5.5 CD63 ELISA

To determine the recovery of intact EVs, the tetraspanin CD63 was detected using a CD63 enzyme-linked immunosorbent assay (Human CD63 ELISA Kit, Thermo Fisher Scientific Inc., Waltham, USA). The CD63 ELISA was performed according to the manufacturer’s instructions, whereby the samples were diluted between 1:2 and 1:10, depending on the protein concentration.

#### 3.5.6 Raman Spectroscopy

Raman spectra were generated with RamanRxn2 (Endress+Hauser GmbH & Co. KG, Rheinach, Switzerland). The bioreactor samples as well as pure culture medium and microcarriers, which were manually suspended in water at the same concentration as in the process, were measured in triplicate with an integration time of 30s.

#### 3.5.7 FTIR Spectroscopy

Cultivation samples and test sets were measured with a ReactIR 702L (Mettler Toledo Inc., Columbus, Ohio, USA). Three measurements were carried out for each sample, in which 32 spectra were recorded and averaged by the measurement software.

#### 3.5.8 SOPAT Microscopy

Using the SOPAT Pa probe, 80 images were taken of each bioreactor sample. Magnetic stirring was used to prevent the microcarriers from settling. In addition, several image series were recorded for every sample, in which the gain, the strobe intensity, the exposure time, and the focus position were varied.

### 3.6 Model Framework

#### 3.6.1 Cultivation of hMSC

The macroscopic kinetic model consists of several monod kinetics and mass balances to describe viable cell density, glucose, and lactate concentration. As in the experiment, cultivation is divided into a growth phase and a collection phase, in which product formation occurs. The kinetics were adapted from previous studies predicting cell growth and product formation of CHO (Chinese hamster ovary) monoclonal antibody process.^113^ To account for cell stress and the higher glucose consumption under cell death, additional kinetics were implemented

1 $$\mu_{d}=k_{d}\frac{Lac}{K_{Dlac}+Lac}+k_{lag\;\max}\frac{K_{M\;lag}}{K_{M\;lag}+t}$$

where *k*_lag max_ and *K*_M lag_ are the respective maximal death rate and monod constant for death under cell stress, respectively. The second term of the death kinetics is therefore time-dependent.

2 $$\frac{dGlc}{dt}=-\left(\frac{\mu -\mu_{d}}{Y_{XGlc}}+m_{Glc}+\frac{q_{product}}{Y_{product\;Glu\cos e}}\right)\times \mathrm{VCD}$$

The glucose consumption was extended by a product-dependent term with the specific particle formation rate *q*_product_ and the product yield coefficient *Y*_product Glucose_.

For estimation, the NL2SOL solver was applied, which reduces the partial least-squares error of the applied model parameters.

#### 3.6.2 Concentration and Buffer Exchange via UFDF

Determination of Blocking Mechanism—During ultrafiltration, a decrease in flux can be caused by concentration polarization, viscosity changes, and changes to the total filtration resistance, which can be caused by different mechanisms.^130^ Similar to normal flow filtration blocking laws,^131^ the analysis of flux decline by regression to power constants can be achieved.^132^

The integral method to fouling analysis, described in detail by Wu,^133^ is applied in this work and consists of 6 main steps, which enables not only the determination of fouling mechanism and blocking constants but also to decide if there is a switch from one mode of fouling to another one.

The appropriate fouling mechanism is integrated in the process model for ultrafiltration/diafiltration based on the model by Grote et al.^134^ The flux is described by the Darcy-Weisbach equation^135^

3 $$J_{v}=\frac{\mathrm{TMP}}{\eta \cdot R}$$

The model has been applied and validated for VLP and LNP similar in size to EVs.^102,103,110,136^ Model parameters were checked to be appropriate for the experimental results.

#### 3.6.3 Chromatographic Purification of EVs

Two general rate models with a lump sum kinetics approach are used to model the chromatography steps.^137,138^ A Langmuir isotherm model is used to describe the equilibrium between the liquid and solid phases.^139,140^ The isotherm parameters of the components depend on the concentration of the modifier.^141^ In the past, it has been shown that this approach is sufficient for model description and optimization in chromatography.^142^

For the simulation of the multimodal size exclusion chromatography, the following adaptations were made: Depending on the size of the components, they can penetrate deeper into the pores of the resin. This effect has an impact on the residence times of the components in the column. To describe this effect, the axial dispersion coefficient (*D*_*ax,i*_), total voidage (ε_tot,*i*_), and pore void fraction (ε_*s,i*_) of the resin are made dependent on the components as represented by the index “*i”.* ^143^

4 $$\frac{\partial c_{i}}{\partial t}=-\frac{u_{\mathrm{int}}}{\varepsilon_{\mathrm{tot},i}}\cdot \frac{\partial c_{i}}{\partial x}+D_{\mathrm{ax},i}\cdot \frac{\partial^{2}c}{{\partial x}^{2}}-k_{eff}\cdot \frac{6}{d_{P}}\frac{\left(1-\varepsilon_{s,i}\right)}{\varepsilon_{S,i}}\cdot \left(\frac{\partial c_{i}}{\partial x}-\frac{\partial c_{p,i}}{\partial x}\right)$$

## 4 Results

### 4.1 Cultivation of hMSC

The cultivation of adherently growing hMSC on a 1 L scale in a stirred tank reactor is divided into two phases: The growth phase and the collection phase (shown in Figure 2a). After reaching at least 3 × 10^5^ cells/mL in the growth phase after 5 days, a media change was performed and the cultivation continued for another 5 days. Harvesting was then performed via depth filtration.

**Figure 2:**
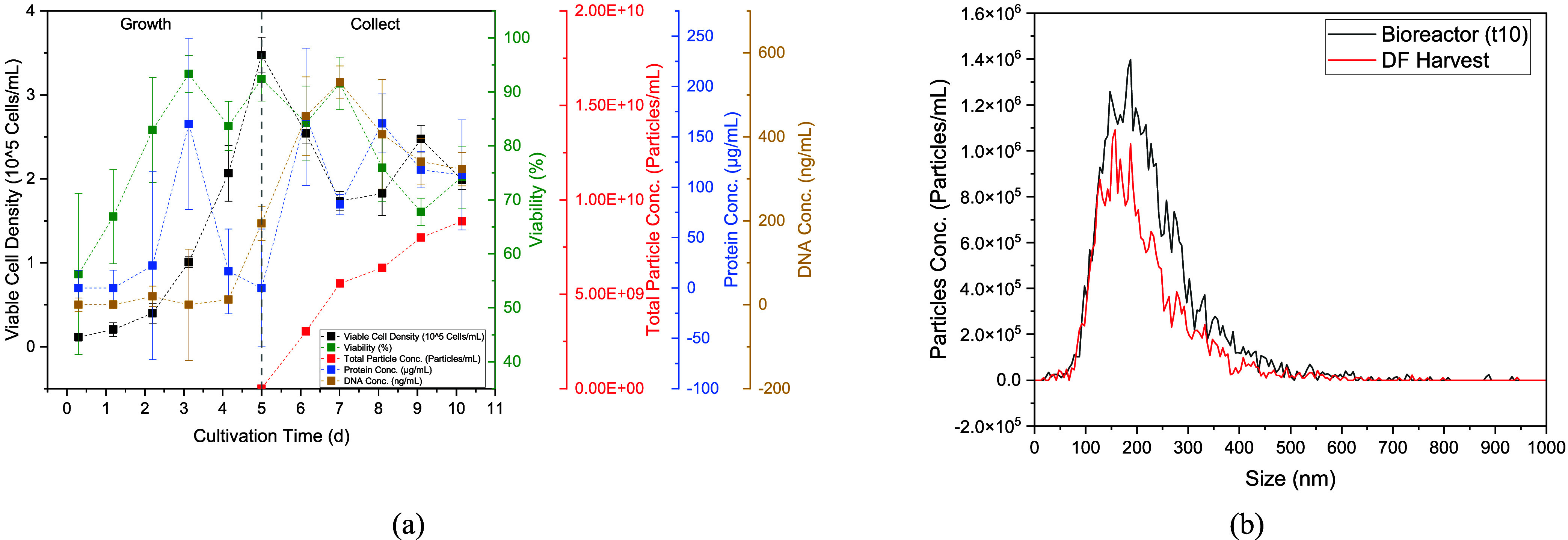
(a) Viable cell density (black dots), total particle concentration (red dots), protein (blue dots), and DNA concentration (yellow dots); (b) particle size distribution measured with NTA of the last day of cultivation (black line) and after harvest by depth filtration (red line).

During the growth phase, no significant change in DNA concentration could be observed. The same applies to the protein concentration, with the exception of feed addition on day three. After the media change, both the protein and the DNA concentration initially rise sharply, which is probably due to the cell death that occurs from day 5 to day 7. The stirring speed was then reduced in order to reduce the shear stress, after which the cell growth increased again. From this point onward, the DNA and protein concentrations also decrease again. Over the entire time of the collection phase, the particle concentration increases constantly up to a final concentration of 8.9 ± 0.09 × 10^9^ particles/mL. Particle concentrations of 4.9 × 10^9^–2.2 × 10^10^ particles/mL are documented for cultivations with the same cell line.^52,66–68,126,144^

The particle size distribution (Figure 2b) is in the range of approximately 50–500 nm, with the peak maximum at the end of cultivation at approximately 180 nm. Depth filtration mainly removes larger particles, so that the peak maximum shifts toward 150 nm.

#### 4.1.1 Process Analytical Technologies

For an automated process, information on key process attributes such as the viable cell density (VCD) or glucose concentration must be available in real time, which can be passed on to the DT and thus enable APC. It is challenging to obtain such information from classic PAT sensors such as conductivity or turbidity in microcarrier cultivations.

The SOPAT Pa probe (resolution: 15–2300 μm) was used to determine the viable cell density. Since the cells grow adherently on microcarriers, it is not possible to image the individual cells as shown for a HEK293 suspension culture.^102^ However, due to cell growth, during which microcarrier aggregates form and metabolites are consumed, or like EVs, released into the culture medium, a change in the image properties can be expected. Analysis was performed with the SOPAT CoCa tool. It applies mathematical operators (“features”) to determine the image properties, such as the gray value, with a total of 15 features available. These and the settings for image acquisition, such as the strobe intensity and gain, as well as the stirrer speed, were then used for statistical evaluation using the commercial program JMP. A simple neural network with two levels and three nodes each was selected as a regression model. With an *R* ^2^ of 0.97, as shown in Figure 3a, the model is already well suited to predict the viable cell density. The number of data points in the low concentration range (up to 0.5 × 10^5^ cells/mL) is significantly higher than at higher concentrations, as can also be seen from the distribution analysis. This is due to the lag phase, which comprises 3 days in the concentration range. In contrast, there is only 1 day in the maximum cell density range. Further training is therefore necessary to optimize the predictive power and increase the robustness of the model. However, it can already be shown that SOPAT offers potential as a PAT tool for predicting the cell density.

**Figure 3:**
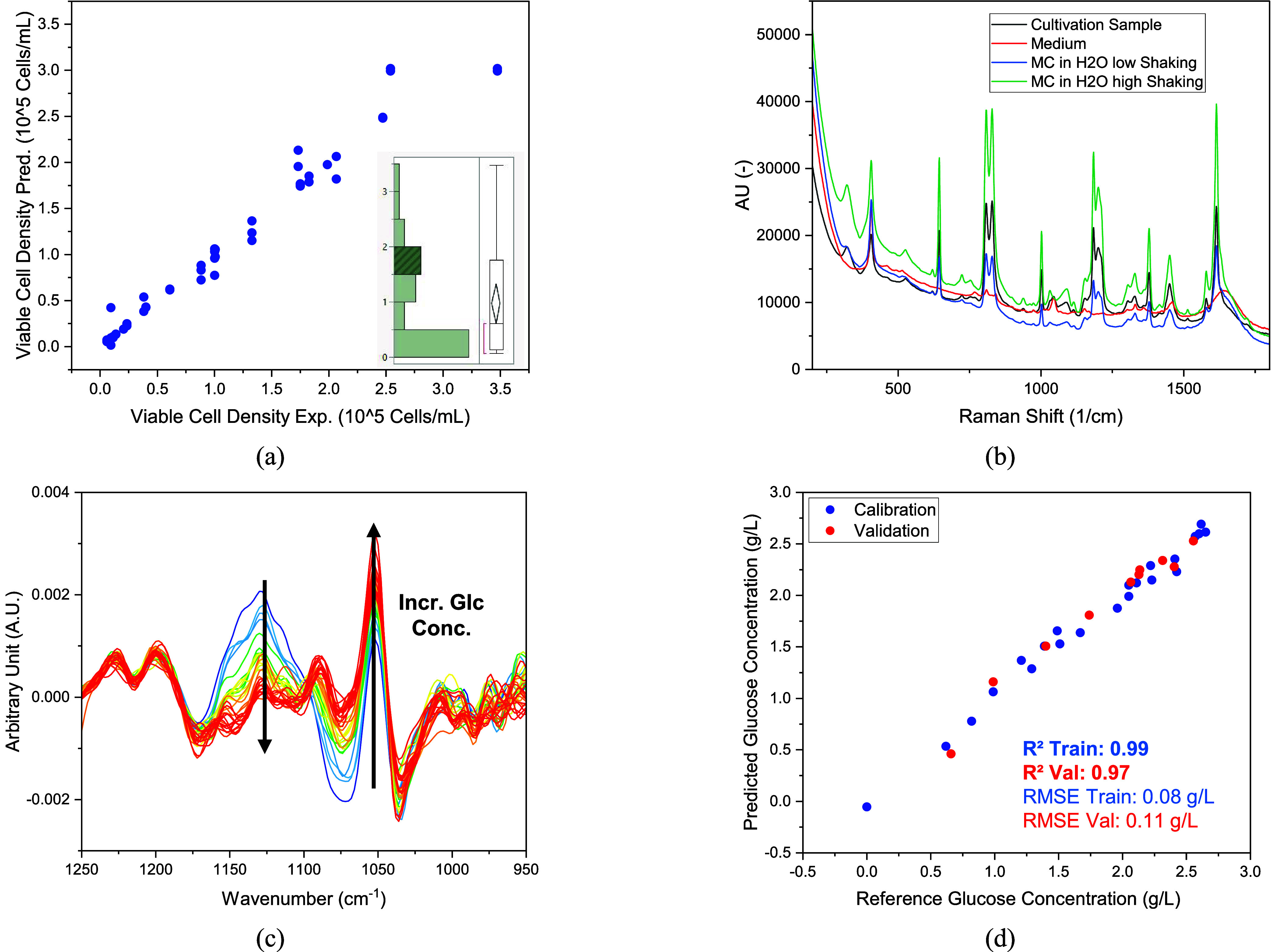
(a) Prediction of VCD with a neural network that utilizes the image information on the SOPAT probe as well as the measurement settings and the stirrer speed, including distribution analysis of the VCD. (b) Raman spectra of the cell culture (black line), cultivation medium (red line), and microcarrier in water at different shaking intensities (blue and green line). (c) Derivative FTIR spectra of the standard and cultivation samples. (d) PLSR results for FTIR spectra for the training data set (blue dots) and the validation data set (red dots).

The detection of critical metabolites such as glucose by spectral analysis is already established in bioprocesses, especially Raman and FTIR.^58,102,110,120^ During cultivation, all samples were measured with these detectors and examined for their applicability.

The intensity of the Raman spectrum (see Figure 3b) is strongly dependent on the stirrer speed (reproduced here offline by manually shaking the sample). In addition, it can be seen that the spectrum of the microcarriers, which are coated with collagen, clearly overlaps that of the medium. Consequently, the use of Raman is not well suited as a PAT sensor in the cultivation of hMSC.

As an alternative method, FTIR was examined in which such a phenomenon could not be observed. Using training sets prepared from the cultivation medium with and without glucose and the cultivation samples, two characteristic peaks could be identified. With increasing glucose concentration, the peak height of the derivative raw spectrum decreases at a wavenumber of 1125 cm^–1^ and increases at a wavenumber of 1055 cm^–1^ (Figure 3c). The entire data set was divided into two subsets, with approximately one-third of the data used for model validation and the remainder for model training. Using partial least-squares regression (PLSR), a good model quality was achieved with a coefficient of determination *R* ^2^ of 0.99 in training and an *R* ^2^ of 0.97. This is underlined by the low RMSE values of 0.08 g/L in training and 0.11 g/L in validation.

### 4.2 Concentration and Buffer Exchange via UFDF

After depth filtration, concentration and diafiltration of the harvest were done by UFDF. This process is divided into an initial concentration with a volumetric concentration factor (VCF) of 2.2, the subsequent buffer exchange with five diafiltration volumes (DV), and a further concentration with a VCF of 2.2.

As shown in Figure 4a, the permeate flux (LMH) first decreases sharply, starting at 89.9 L/m^2^/h and then approaches a constant flux of approximately 20 L/m^2^/h. On average, a permeate flux of 25.2 L/m^2^/h is achieved, which is also found in Zakhem et al. (25.8 L/m^2^/h),^125^ whose process parameters were used as a basis for the UFDF step in this study. In addition, a comparable recovery of 88.3 ± 4.6% (75.1–91.6%) and protein removal of 70.8 ± 3.0% (48–74.2%), as well as DNA reduction of 77.1 ± 8.8% (21–85%)^67,125^ can be achieved. At the same time, no particles can be detected in the permeate (see Figure 4b), an indication of complete product retention.

**Figure 4:**
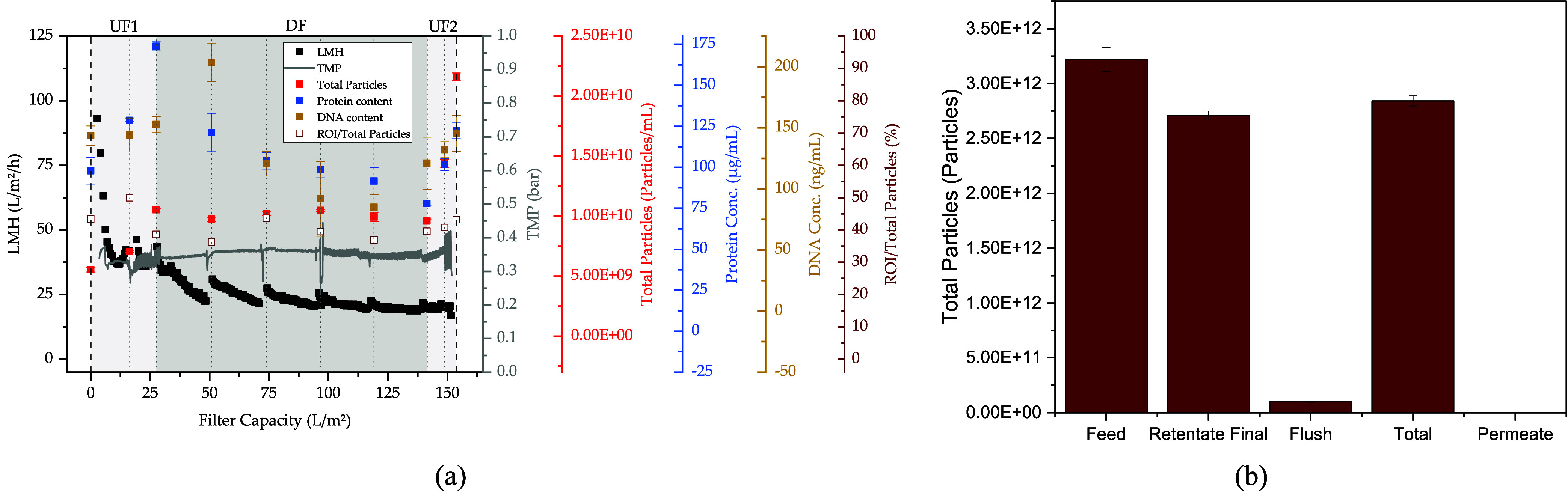
(a) Permeate flux (LMH, black dots), TMP (gray line), total particle (red dots), protein (blue dots), and DNA concentration (yellow dots) by filter capacity, (b) Total particles in the feed, retentate, flush, and permeate.

A summary of the process parameters as well as the recovery and removals achieved is shown in Table 1.

**Table 1:** Process Characteristics of the Concentration and Buffer Exchange Steps via UFDF

Membrane Area (cm^2^)	115
Particle Load (Particles/m^2^)	2.13 × 10^14^
Volume Load (L/m^2^)	50.6
Shear Rate (s^–1^)	4000
TMP (bar)	0.34
Average Flux (L/m^2^/h)	25.2
Recovery (%)	88.3 ± 4.6
Protein Removal (%)	70.8 ± 3.0
DNA Removal (%)	77.1 ± 8.8

### 4.3 Chromatographic Purification of EVs

#### 4.3.1 Multimodal Size Exclusion Chromatography

Due to size exclusion, particles larger than 400 kDa are present in the flow through. This includes extracellular vesicles as well as some genomic DNA (90–1000 kDa).^145^ Molecules that fall below the size exclusion limit of 400 kDa can diffuse into the pores. Due to the functionalization of the beads, negatively charged and hydrophobic species are bound, and all others are separated due to their size. Binding of HCPs in particular is to be expected, as these are expected to be negatively charged.^146–148^

Since the EVs are present in the flow through, no selective elution of bound species is necessary. One CV of the UFDF retentate was loaded onto the Capto Core 400, and after the washing step, a CIP step was carried out in reverse flow according to the column manufacturer’s instructions. The chromatogram of purification is shown in Figure 5.

**Figure 5:**
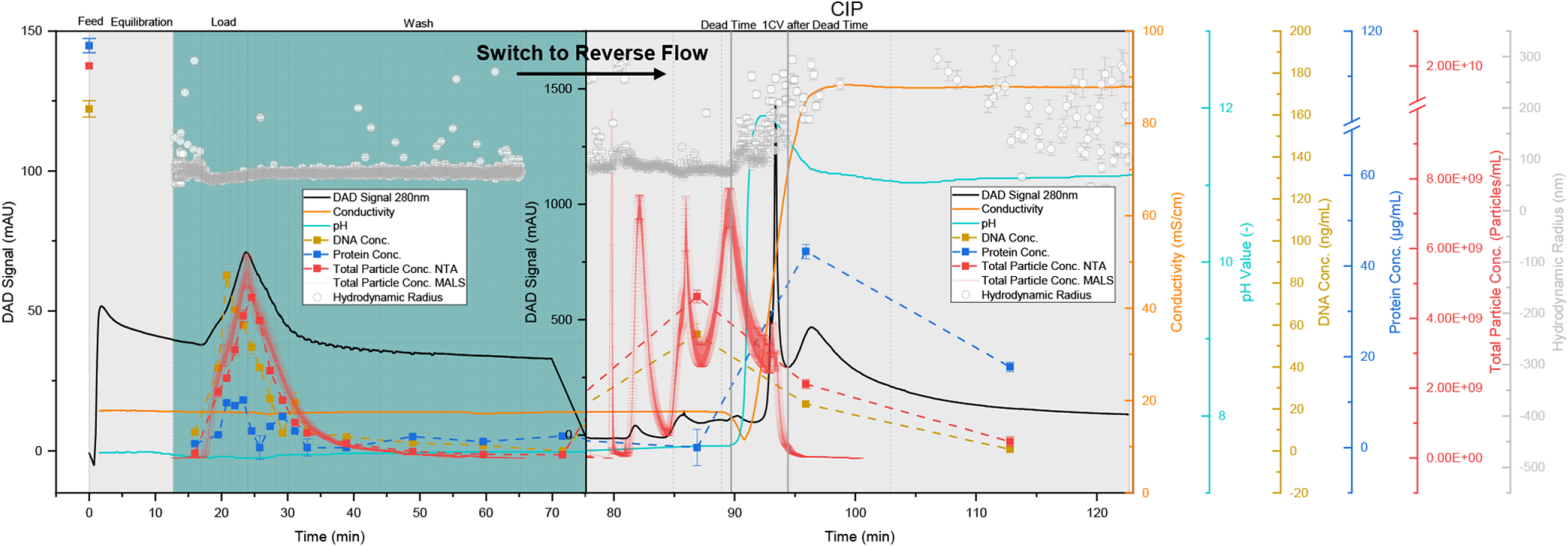
Purification of 1 CV UFDF retentate with multimodal Capto Core 400. (a) Chromatogram with DAD signal at 280 nm (black line), conductivity (orange line), pH value (light blue line), total particle concentration (red line) and hydrodynamic radius (gray dots) obtained from MALS/DLS detector, total particle concentration measured by NTA (red dots), DNA concentration (yellow dots), total protein concentration (dark blue dots), pooled product fractions are highlighted in light blue, dashed vertical lines indicate fractionation.

As expected, the majority of the proteins elute in the CIP step, which leads to a reduction of 82.0 ± 1.2% of total protein and is consistent with the 79% removal of proteins achieved using Capto Core 400 in the literature.^126^ This results in a concentration of 2.43 ± 0.13 μg/mL total protein in the flow through and wash fractions, which is below the regulatory threshold of 100 μg/mL.

Due to their size, DNA is also present in the flow through, which is why the removal of DNA is significantly lower with 37.2 ± 2.3% (104.6 ± 3.9 ng_DNA_/dose). The limit of dsDNA per dose, which has been set by the regulatory authorities to <10 ng_DNA_/dose^149^ for use in cell and gene therapy, is not met, and an additional purification step is necessary.

In contrast to Jung et al., who achieved a recovery of 97% with a load of 1 CV,^126^ only a total of 37.7 ± 1.6% of all particles could be recovered after NTA and 44.3 ± 7.4% after MALS in the flow through and wash fractions. A loss of approximately 50% of the particles was also previously observed by other working groups for extracellular vesicles^150^ and lentivirus-like particles^151^ during purification using Capto Core 700. The reason given for this was a possible retention of the particles within the frit.

This assumption can be supported by the evaluation of the CIP step using MALS. As this is carried out in reverse flow, elution of bound particles is only to be expected after the dead time of 9.4 min, which the CIP buffer requires to flow through the column completely. In the chromatogram shown in Figure 5, the elution of the remaining particles in the CIP step can be seen. Four peaks are observed in the MALS, whereby the first three peaks are within the dead time of 1.7, 5.6, and 9.2 min. These particles are presumably retained by the frit and possibly by the column. Only the last and smallest peak with 2.9 ± 0.7% of all particles occurs at the time the CIP buffer reaches the outlet of the column and can be attributed to bound particles or DNA.

#### 4.3.2 Anion Exchange Chromatography

As a possible alternative to the Capto Core 400, separation was carried out by using a strong anion exchanger. Since purifications via AEX with prior DNase treatment are mainly found in the literature,^1,42,94,152,153^ two runs were conducted, one in which the UFDF retentate was treated with DNase before loading (Figure 6 on the right) and one untreated (Figure 6 on the left) was loaded onto the column.

**Figure 6:**
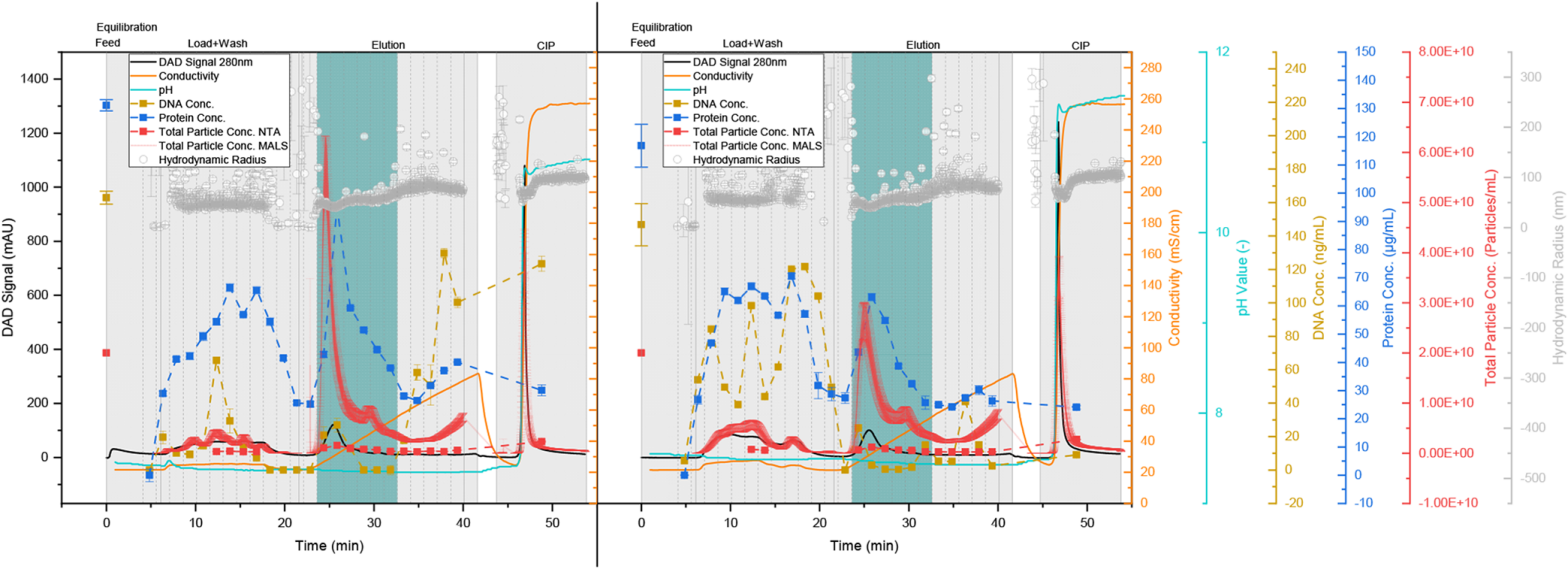
Purification by AEX of 10 CV UFDF retentate treated with DNase (right) and without DNase treatment (left). Chromatogram with DAD signal at 280 nm (black line), conductivity (orange line), pH value (light blue line), total particle concentration (red line), and hydrodynamic radius (gray dots) obtained from MALS/DLS detector, total particle concentration measured by NTA (red dots), DNA concentration (yellow dots), total protein concentration (dark blue dots), pooled product fractions are highlighted in light blue, dashed vertical lines indicate fractionation.

Differences can be seen primarily in the elution behavior of the DNA. Contrary to expectations, the addition of DNase did not significantly reduce the DNA concentration in the feed (from 163 ± 4 to 147 ± 13 ng/mL). One possible reason for this could have been a nonideal reaction environment due to salt in the buffer. Nevertheless, the courses of the DNA concentration differ: when treated with DNase, hardly any DNA binds and the bound amount elutes primarily at a low salt concentration (<30 mS/cm). Without pretreatment, on the other hand, hardly any DNA is present in the flow through and elutes at salt concentrations >50 mS/cm. As shorter DNA (strands) bind less well to the AEX resin,^147^ this indicates that although the treatment with DNase did not achieve complete digestion, it did reduce the size of the DNA. Based on the observed elution behavior, a comparable removal of 94.9 ± 1.1% (without pretreatment) and 95.8 ± 1.0% (with DNase) is achieved, resulting in 7.48 ± 1.36 ng_DNA_/dose and 7.56 ± 1.03 ng/dose, respectively, which is below the critical concentration of 10 ng_DNA_/dose.

The protein reduction is also comparable with 67.5 ± 2.7% (without DNase) and 69.8 ± 2.3% (with DNase), which corresponds to a concentration of 43.33 ± 0.27 and 43.05 ± 0.27 μg/mL, respectively. The set conductivity of 23 mS/cm reduces the binding of proteins to the matrix,^1^ so that a large proportion of the proteins can already be found in the flow through, as expected.

However, a difference can be seen in the height of the elution peak, where the particle concentration, according to MALS, without pretreatment with DNase is approximately twice as high as with DNase. Due to the 2 h incubation at 37 °C with DNase, a loss of approximately 17% of particles in the feed was observed, which consequently also reduced the total amount of loaded particles. In addition, the yield of 49.3 ± 8.5% is slightly lower compared to 55.1 ± 6.8% after MALS. Nevertheless, both are in the range of the recovery documented in the literature for the purification of extracellular vesicles using Poros HQ and with pretreatment with DNase (53.9 ± 15.9%).^1^ In combination with the lower concentration in the feed, this results in a total particle recovery of 9.0 ± 1.3 × 10^10^ particles (with DNase) versus 12.1 ± 1.3 × 10^10^ particles (without DNase) in the product fractions.

#### 4.3.3 Combination of Multimodal SEC and AEX

In order to investigate the assumption made above on the retention of particles in the frit of the Capto Core 400, three times the amount (3 CV) of UFDF retentate was loaded onto the column. The pooled product fractions were then loaded onto the Poros HQ in order to take advantage of the improved removal of DNA. In addition, it was investigated whether the recovery in the AEX can be improved by removing smaller particles and proteins during the Capto Core 400 step.

The resulting chromatogram of Capto Core 400 is shown in Figure 7a. Due to the higher load, the particle concentration in the flow through is already approximately three times higher than the 1 CV load. In contrast, peaks one to three run into each other within the CIP step and are no longer separated. However, the maximum height of the peak is comparable to the first experiment. This is also reflected in the total number of particles in the CIP. At 1 CV load, 3.80 ± 0.69E10 particles, and at 3 CV load, on average, 2.77 ± 0.11 × 10^10^ particles are found in the CIP. As the majority of the particles in the CIP elute within the dead time even at higher loads, it can be assumed that the frit is saturated at around 3 × 10^10^ particles and that the proportional loss decreases with a higher load.

**Figure 7:**
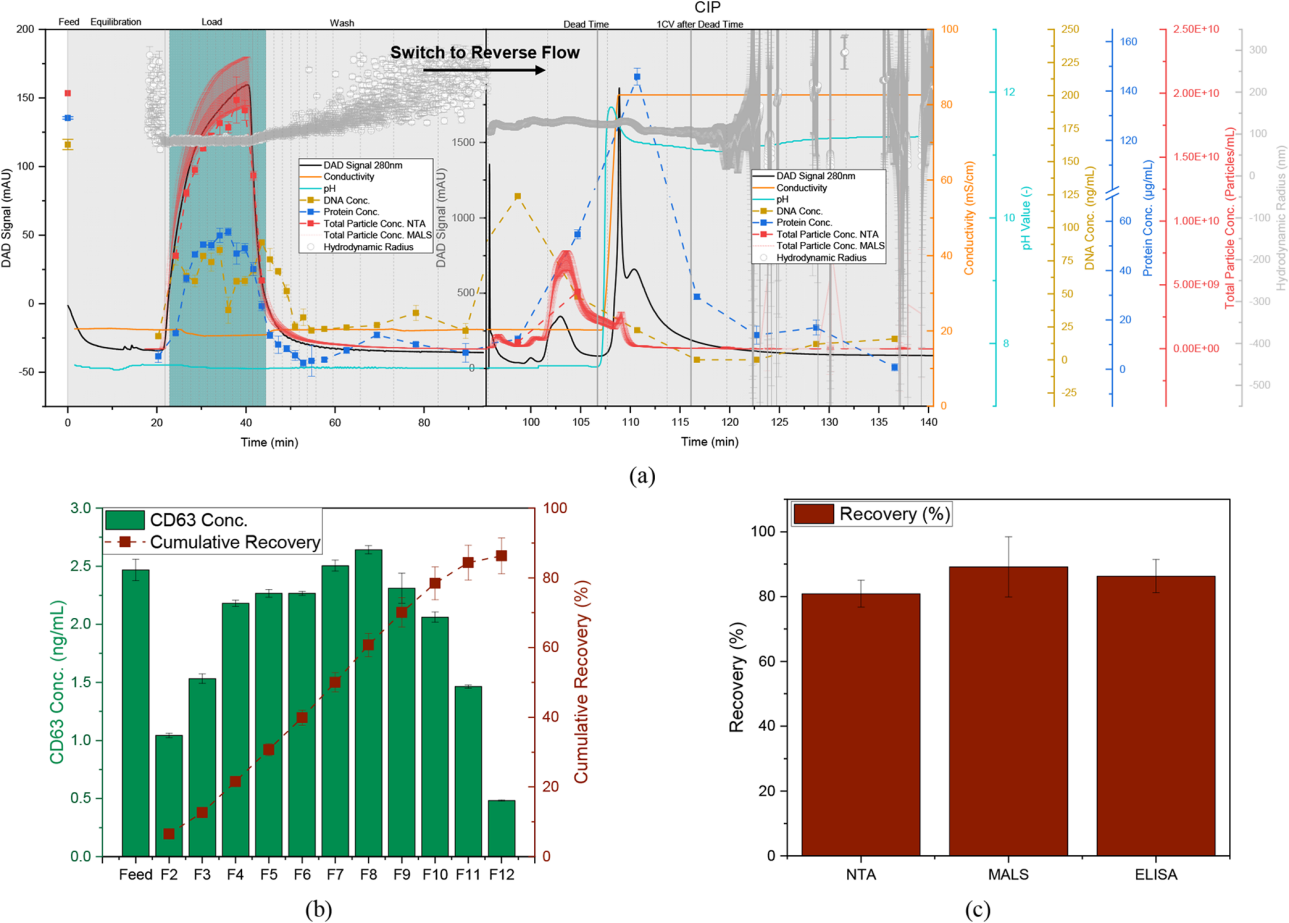
Purification of 3 CV UFDF retentate with multimodal Capto Core 400. (a) Chromatogram with DAD signal at 280 nm (black line), conductivity (orange line), pH value (light blue line), total particle concentration (red line), and hydrodynamic radius (gray dots) obtained from MALS/DLS detector, total particle concentration measured with NTA (red dots), DNA concentration (yellow dots), total protein concentration (dark blue dots), pooled product fractions are highlighted in light blue, dashed vertical lines indicate fractionation. (b) CD63 concentration and cumulative recovery of product fractions measured by ELISA. (c) Total recovery in pooled product fractions.

The recovery can thus be increased to 89.2 ± 9.3% (MALS) and 80.1 ± 4.1% (NTA) (see Figure 7c). Since both MALS and NTA only provide information on the recovery of total particles, an ELISA was additionally performed to detect the tetraspanin CD63 of the EVs. In addition to CD81 and CD9, this is the most used marker^154^ in the cell line used with Rooster Collect EV as collection medium.^52,67,68,101,126,152^ The concentration curves and the cumulative recovery in the product fractions are shown in Figure 7b. According to ELISA, 86.3 ± 5.1% of the EVs can be recovered in product fractions two to 12, which were collected from 22.8 to 44.5 min. Due to the comparable recoveries via the determination of the total particle concentration using MALS and NTA, it can be assumed that almost all of the recovered particles are EVs, which is consistent with the analysis by Jung et al.^126^ using a positive membrane dye.

However, the higher loading reduces the removal of total protein to 62.9 ± 1.8%. Nevertheless, no breakthrough is observed, so that the binding capacity is probably not reached, and the additional protein in the flow through may be related to the product and protein aggregates.

Since, in contrast to loading with 1 CV, the entire flow through and wash do not serve as product fractions and, in particular, more DNA is washed out in the wash step, the DNA removal can be increased to 47.9 ± 4.5% and the dose-related DNA can be reduced to 42.27 ± 2.71 ng_DNA_/dose.

The product fractions from the Capto Core 400 separation were then loaded onto the Poros HQ. Two repetitions were carried out, with 12 CV being loaded in both cases. The corresponding chromatogram is shown in Figure 8. Despite the lack of pretreatment with DNase, the majority of the DNA is in the flow through. However, bound DNA elutes primarily at conductivities >50 mS/cm in the gradient or in the CIP step. Thus, 95.8 ± 1.7% of the DNA can be eliminated again, resulting in 5.08 ± 0.71 ng_DNA_/dose. Thus, the dose-specific amount of dsDNA can be further reduced, resulting in a safer process.

**Figure 8:**
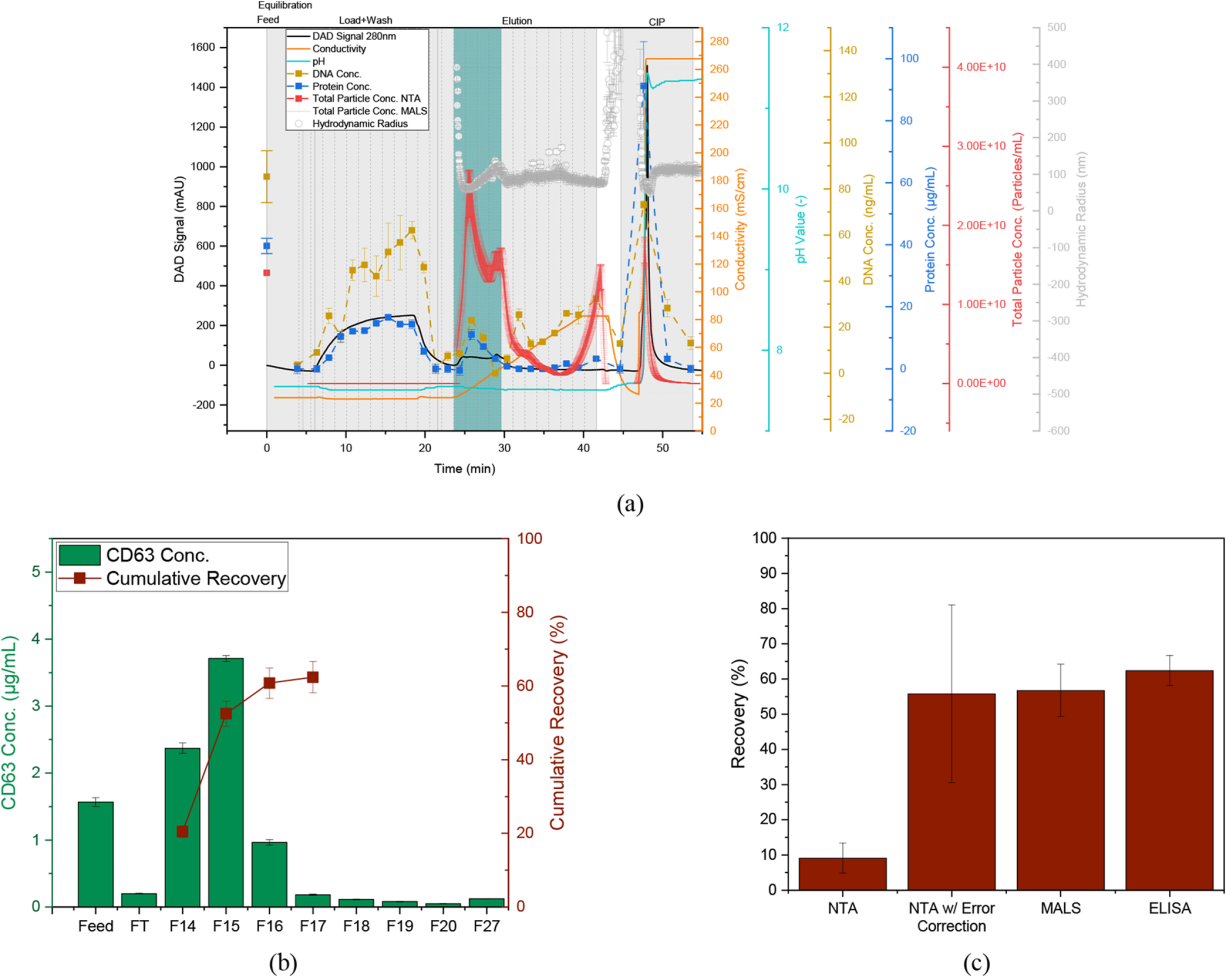
Purification of 12 CV pooled Capto Core 400 fractions with AEX. (a) Chromatogram with DAD signal at 280 nm (black line), conductivity (orange line), pH value (light blue line), total particle concentration (red line), and hydrodynamic radius (gray dots) obtained from MALS/DLS detector, total particle concentration measured by NTA (red dots), DNA concentration (yellow dots), total protein concentration (dark blue dots), product fractions are highlighted in light blue, and dashed vertical lines indicate fractionation. (b) CD63 concentration and cumulative recovery of the product fractions measured by ELISA. (c) Total recovery in pooled product fractions.

In addition, the concentration of proteins in the elution fractions decreases and either does not bind or is eluted in the CIP, so that 92.9 ± 1.3% of the total protein is removed.

As in the previous experiments, the EVs elute at the beginning of the gradient, whereby the product fractions from 23.6 to 29.6 min (F14–17) were selected. This was done on the basis of the ELISA results (Figure 8c), according to which there is no significant increase in recovery after fraction 17. In addition, it can be seen that although approximately 20% of all particles are recovered in the CIP step, only 2.3 ± 0.1% of CD63 is detected here. Consequently, an additional separation of intact tetraspanin-positive EVs from nonintact tetraspanin-negative particles and other extracellular vesicles as well as DNA takes place. It also explains the slightly lower recovery determined with MALS, as MALS can only measure the total particle number. The recovery is 56.8 ± 7.4%, whereas the ELISA recovery is 62.4 ± 4.3% (see Figure 8c). Comparable results to those obtained with the MALS were to be expected with the NTA. However, this was only 9.1 ± 4.2%. During the measurements, however, it was observed that the particle concentration increased by approximately 25% during longer measurement times and that the deviation was 40% in repeated measurements. Possible reasons for the increase during a measurement could be aggregates that have formed in the elution buffer and dissolve during the measurement and thus reenter the focus area of the NTA. The deviation between the measurements was observed in particular after short-term storage in the refrigerator at 4 °C. This could be due to a degradation of the particles in the buffer. A loss of 20–40% when stored in phosphate buffers for one to 4 days at 4 °C was also observed by van de Wakker et al.^3^ If these errors are taken into account when calculating the recovery, a corrected recovery of 55.8 ± 25.3% is obtained, which is then in the range of the MALS and ELISA.

### 4.4 Process Summary

In order to be able to quantify the success of the purification, the purity of the EVs, which is typically determined by the total number of vesicles in relation to the total amount of protein, is shown in Table 2 and Figure 9b, in addition to the absolute concentrations. Furthermore, the amount of DNA per dose was determined, as this is required by regulatory standards and must not exceed the limit value of 10 ng_DNA_/dose.^149^ A dose of 1 × 10^10^ particles of EVs was assumed for this purpose.^12^ By combining the multimodal SEC (CC400) and the AEX (Poros HQ), the purity of the EVs can be increased by a factor of 50 from an initial 5.6 × 10^10^ particles/mg_Protein_ to 2.7 × 10^12^ particles/mg_Protein_ and is thus at the upper limit of the range of 3.0 × 10^11^–4.0 × 10^12^ particles/mg_Protein_ described in the literature.^1,67,126,152,155^ In addition, the EV purity is thus on average 10 times higher than that with a single chromatography purification step. This is due to the efficient utilization of the advantages of the CC400 and the Poros HQ. As can be seen from Table 3, the removal of proteins in the CC400 is higher with 82% than in the Poros HQ, with an average of 69%. Although this falls to the level of the AEX due to the higher loading in the combination of the two chromatography steps, possibly due to product-related proteins, it enables more efficient removal in the AEX, as the lower protein concentration results in a lower amount being coeluted.

**Figure 9:**
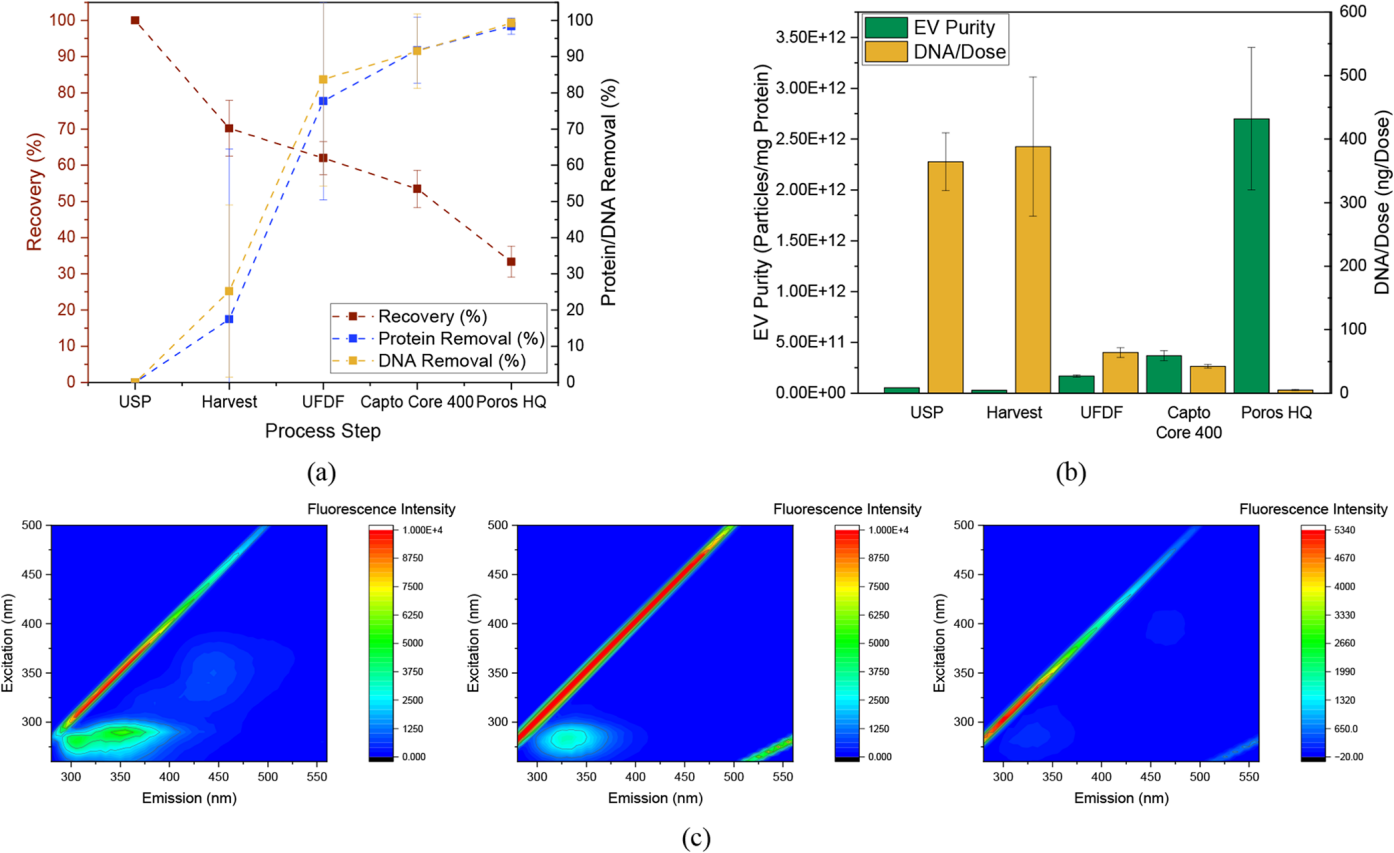
(a) Total recovery of EVs (red points) as well as total removal of proteins (blue points) and DNA (yellow points) over the process steps. (b) EV purity in EVs/mg_Protein_ (green bars) and ng_DNA_/dose (yellow bars) over the process steps. (c) 2D fluorescence of harvest (left), UFDF retentate (middle), and AEX product fractions (right).

**Table 2:** Concentrations of Particles and Impurities (Proteins and DNA) as well as Characteristic Purity Numbers (Particles/mg_Protein_ and ng_DNA_/dose)

Process Step		Particle Conc. (×10^10^ Part./mL)	Protein Conc. (μg/mL)	EV Purity (Part./mg_Protein_)	DNA Conc. (ng/mL)	DNA/Dose (ng_DNA_/dose)
Cultivation		0.89 ± 0.01	112.2 ± 54.5	5.6E10	323.1 ± 39.8	364.5 ± 45.4
Depth Filtration		0.6 ± 0.06	97.8 ± 8.0	2.9E10	233.0 ± 36.3	388.4 ± 109.3
UFDF		2.00 ± 0.003	116.8 ± 1.6	1.7 ± 0.1E11	163.4 ± 4.0	64.1 ± 7.7
CC400	1 CV Load	0.10 ± 0.008	2.4 ± 0.1	4.2 ± 0.6E11	10.7 ± 0.4	104.6 ± 3.9
Poros HQ	w/o DNase	1.35 ± 0.14	43.3 ± 0.3	3.1 ± 0.4E11	10.1 ± 1.8	7.5 ± 1.4
	w/DNase	1.00 ± 0.15	43.1 ± 0.3	2.3 ± 0.4E11	7.6 ± 1.0	7.6 ± 1.0
Combination	CC400 (3 CV)	1.55 ± 0.13	41.5 ± 1.9	3.7 ± 0.5E11	68.9 ± 4.5	42.3 ± 2.7
	Poros HQ	1.48 ± 0.15	5.5 ± 0.1	2.7 ± 0.7E12	7.5 ± 0.9	5.1 ± 0.7

**Table 3:** Recovery of Particles and Removal of Proteins and DNA for Every Process Step

		Particle Recovery (%)				
Process Step		NTA	MALS	ELISA	Protein Removal (%)	DNA Removal (%)
Cultivation		-	-	-		
Depth Filtration		70.2 ± 7.7			17.5 ± 47.0	25.2 ± 23.8
UFDF		88.3 ± 4.6	-	-	70.8 ± 3.0	77.1 ± 8.8
CC400	1 CV Load	37.7 ± 1.6	44.3 ± 7.4		82.0 ± 1.2	37.3 ± 2.3
Poros HQ	w/o DNase		55.1 ± 6.8		67.5 ± 2.7	94.9 ± 1.1
	w/DNase		49.3 ± 8.5		69.8 ± 2.3	95.8 ± 1.0
Combination	CC400 (3 CV)	80.9 ± 4.1	89.2 ± 9.3	86.3 ± 5.1	62.9 ± 1.8	47.9 ± 4.6
	Poros HQ	9.1 ± 4.2 (55.8 ± 25.2)	56.7 ± 7.4	62.4 ± 4.3	92.9 ± 1.3	95.8 ± 1.2

In contrast, because of its size, the removal of DNA is not as successful as with the AEX, which enables a removal of >94% of the DNA, compared to the CC400 with 37–48%. The DNA reduction in the AEX remains constant during the purification of the CC400 product pool, so that the amount of DNA can be reduced to 5.1 ng_DNA_/dose, which fulfills the regulatory requirements. Although the limit of 10 ng_DNA_/dose is also not exceeded in a single AEX step, it is higher at approximately 7.5 ng_DNA_/dose and is in addition at the upper end of amount reported in the literature (3.5–9.0 ng_DNA_/dose).^1,156,157^ Consequently, with the preliminary CC400, a safer process can be achieved that meets the regulatory requirements and is less sensitive to fluctuations in the process.

If the recoveries are considered, comparable recoveries can be determined with in-line MALS/DLS and ELISA. It should be noted that MALS measures the total particle concentration, like NTA, whereas ELISA detects the product-specific tetraspanin CD63. This results in slightly higher yields after AEX via the ELISA than measured with the MALS, as depletion of nonintact EVs is possible here.^107^ However, the ELISA fails during the first process steps because, without prior complex sample preparation, the measurement can be biased by free protein. Since larger particles are separated in the depth filter during harvest, the recovery of product in this process step is probably higher than the NTA determined.

In contrast to the established NTA method, MALS has the advantage that it can be directly integrated into the process and is therefore available as a PAT detector. Further advantages also apply to unstable samples, as shown after AEX. For lentiviruses, it has previously been shown that elution with salt can lead to degradation.^105^ In addition, aggregation during storage can influence the measurement using NTA.^3^ Consequently, fast subsequent handling is essential to transferring the particles into a stabilizing environment.

The resulting yields and purities for the process steps are shown in Figure 9a. Overall, a recovery of 33.4% can be achieved, which is comparable with the documented values of 4–60%.^1,42,67,68,144,152^ At the same time, 98.4% (literature: 96.6–99.2%)^1,67,152^ of all proteins and 99.3% (literature: 95.3–99.5%)^1,67,152^ of the DNA are removed. The progress of the purification can also be qualitatively tracked by using 2D fluorescence (Figure 9c). The diagonal represents the excitation wavelength that is inherently measured in the emission spectra. Proteins are known to have an absorption maximum at an excitation wavelength of 280 nm, resulting in emission in the range of approximately 300–400 nm.^158,159^ DNA is often considered to be nonfluorescent, but it was found that DNA also emits slightly at this wavelength.^160^ The intensity at 300–400 nm (emission) decreases significantly over the process so that after the AEX (Figure 9c, right), hardly anything is detected in this range, which matches the purification achieved. Due to second-order Rayleigh scattering, a signal at an emission wavelength of >500 nm at twice the excitation wavelength can be detected. The high proportion of proteins and DNA and the lower particle concentration after cultivation (Figure 9c, left) result in no detectable fluorescence. The UFDF (Figure 9c, middle) removes impurities and concentrates the particles so that Rayleigh scattering is clearly detectable in this area. In the further purification process, the intensity decreases due to the expected yield losses. In contrast to the impurities, however, the scattering is still clearly visible.

As a final quality control, the biofunctionality of the produced EVs should be demonstrated. Kusuma et al. reported that the transformation from a 2D to a 3D spheroid-based hMSC culture could lead to a reduced immunomodulatory function.^161^ However, it has previously been shown that 3D spheroid-derived EVs produced with the cell line used in this study showed consistent biofunctionality compared to 2D culture.^49,51,52,162^ Furthermore, the preservation of this was also demonstrated for purified EVs.^67,125,152^ It can therefore be assumed that the functionality of the EVs purified in this study is also maintained.

In summary, an efficient, scalable process was developed, resulting in EVs that are sufficiently pure to fall below the regulatory threshold in cell and gene therapy products of 10 ng_DNA_/dose^149^ dsDNA. Since the total protein concentration at the end of the process is only 5.5 μg/mL, it can also be assumed that the typical limit for biologics of <100 μg/mL (100 ppm)^12^ of HCP is also met.

### 4.5 Digital Reproduction of Experimental Results

#### 4.5.1 Cultivation of hMSC

Modeling of the cultivation was performed with a macroscopic kinetic model adapted from a CHO process.^113^ Parameters that were experimentally difficult to determine, like Monod constants and maintenance coefficients, were adapted initially from the CHO cell model, as hMSCs are also mammalian cells and should exhibit enzymes with similar kinetic behavior. Yield coefficients and maximal growth rates were calculated from experimental data.^163^ The modeling procedure was divided into two phases (growth and collect) as in the experiment, each with a unique parameter set. While in the first phase growth and consumption rates exhibit typical behavior for a batch process,^164,165^ the second phase can be characterized by a higher degree of cell stress, which resulted in higher lactate levels and cell death while product formation was induced.

The model describes cell death by a high level of ammonia and lactate. As with the media exchange, those metabolites were depleted; a prediction of cell death therefore was not feasible with the current model implementations. To account for cell death at the beginning of the collect phase, an additional stress term was implemented, describing a lag phase kinetic within the death term. This adjustment was deemed feasible, as cells undergo metabolic adjustments when exposed to varying media components. Additionally, the simple kinetics of the applied model only suggests consuming glucose under cell death by the cell’s maintenance coefficient. Within the second phase of the cultivation, the cell’s glucose consumption, however, is much higher than that under maintenance, leading to the assumption that the media components inducing exosome production lead to higher uptake rates even under cell death. To align the model with the experimental data, an additional yield coefficient product/glucose is introduced for the latter half of the cultivation.

Table 4 presents the critical model parameters used to predict the hMSC cultivation process. Monod constants and maintenance coefficients were adapted from the CHO process, while product-related kinetics and stress term parameters were defined only for the second cultivation phase. Experimental determination of yield coefficients and maximal growth rates provided results in accordance with existing literature.^164,165^ Notably, the yield coefficient for lactate/glucose exceeds the value of one, indicating that it should be interpreted as an apparent value that compromises growth-related glucose consumption as well as other substrates that are metabolized into lactate not covered in detail by this model.

**Table 4:** Model Parameters Used for the hMSC Cultivation Prediction. Values marked by an asterisk indicate additional estimation during the collect phase to enhance the models’ predictive power

Parameter	Growth phase	Collect phase	Unit
μ_max_*	0.039	0.036	h^–1^
*k*_D_	0.004	0.004	h^–1^
*K*_Glc_	1	1	mmol/L
*K*_I Lac_	43	43	mmol/L
*K*_D Lac_	45.8	45.8	mmol/L
*m*_Glc_	0.07	0.07	mmol_Glc_/10^6^cells*h
*Y*_X Glc_*	0.038	0.031	10^6^cells/mmol_Glc_
*Y*_Lac Glc_*	1.68	2.11	mmol_Lac_/mmol_Glc_
*q*_product_	n/a	5.3 × 10^–13^	mmol_product_/10^6^cells*h
*Y*_Product Glc_	n/a	1.0 × 10^–12^	mmol_product_/mmol_glucose_
*k*_lag max_	n/a	0.04	h^–1^
*K*_M lag_	n/a	160	h^–1^

In the collection phase, parameters such as maximal growth rate and yield coefficients were adjusted to a minor degree to enhance the predictive accuracy under cell death conditions. These modifications attest to the precision and reliability of the experimental data.

Figure 10 shows the results of hMSC Fed-Batch modeling with the applied model parameters from Table 4. In the growth phase, VCD, glucose, and lactate could be predicted accurately, while in the collect phase, VCD is predicted in a trendwise manner but did not alter the model’s accuracy to predict the metabolites glucose and lactate as well as exosome production. The newly introduced stress term parameters as well as the product-related yield coefficient effectively enhanced the predictive power of the model. A higher Y_Lac/Glc_ also indicates increased cell stress, which is coherent with media exchange in the second half of cultivation.

**Figure 10:**
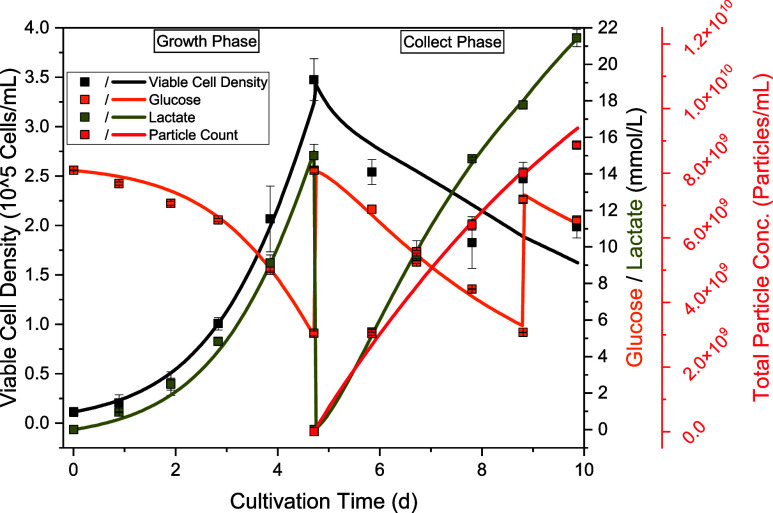
Cultivation of exosomes with hMSCs. Dots represent experimental data, and solid lines represent the respective simulation runs.

Overall, the simple macroscopic model can predict not only viable cell density but also critical metabolites like glucose and product formation accurately, which can be beneficial when incorporating the model into a process control environment. In the growth phase, a minimal viable cell density of 3 × 10^5^ cells/mL is needed in order to achieve a sufficient productivity during the collect phase. With SOPAT as real-time measurement of VCD, the model can determine the optimal time point to proceed to the collect phase while monitoring glucose levels with FTIR. With the calibrated and offline verified (SEC-MALS) titer prediction, the process model can serve as a soft sensor so that optimal process conditions can be determined and hold times before the following downstream process can be eliminated.

#### 4.5.2 Concentration and Buffer Exchange via UFDF

Following the integral method to fouling analysis (cf. Table 5) results in identifying the intermediate pore blocking with allowance for crossflow removal to be the dominant mechanism in the initial phase of filtration, with a coefficient of regression of *R* ^2^ = 0.9583 before the infliction point at approximately 11 L/m^2^. After the infliction point with *R* ^2^ = 0.84, cake filtration is dominant.

**Table 5:** Identification of the Fouling Mechanism during the UFDF Step with Corresponding Blocking Constants

Fouling index	Kn	JR
*n* = 1 (Intermediate)	0.18175	15.995
*n* = 0 (Cake)	0.00195	20.0513

Implemenation of these fouling mechanisms in the process model enables accurate description over the different stages of initial concentration, buffer exchange, and final concentration as shown in Figure 11.

**Figure 11:**
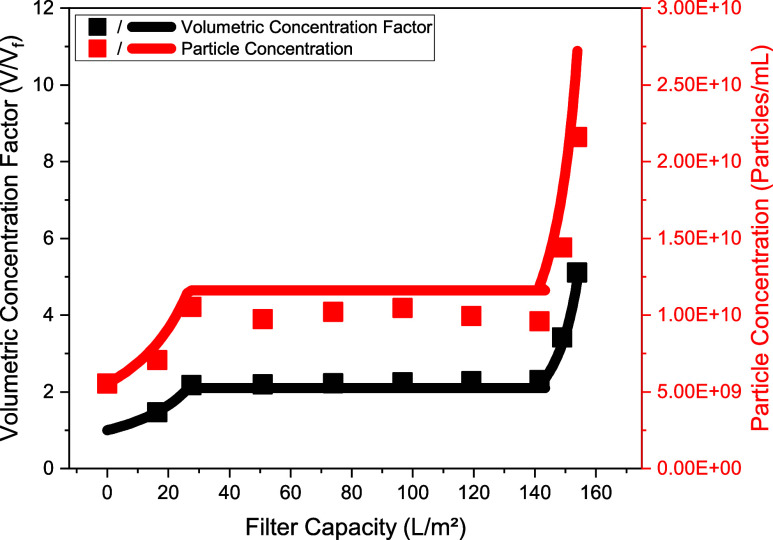
Concentration change during initial concentration, buffer exchange, and final concentration. Points are experimental data, lines are process model simulations.

#### 4.5.3 Chromatographic Purification of EVs

As previously done,^103^ the fluid dynamic model parameters *D*_ax_, total porosity, and pore porosity are determined via the salt gradient of the separation. Offline analysis is used as a basis to determine the isotherm parameters and kinetics parameters. Both the concentration in the individual fractions and the shape of the peak are matched. This is shown in Figure 12 for the MM-SEC and in Figure 13 for the AEX. The determination is limited to the components in the relevant range, i.e., the pool and the components eluting before and after.

**Figure 12:**
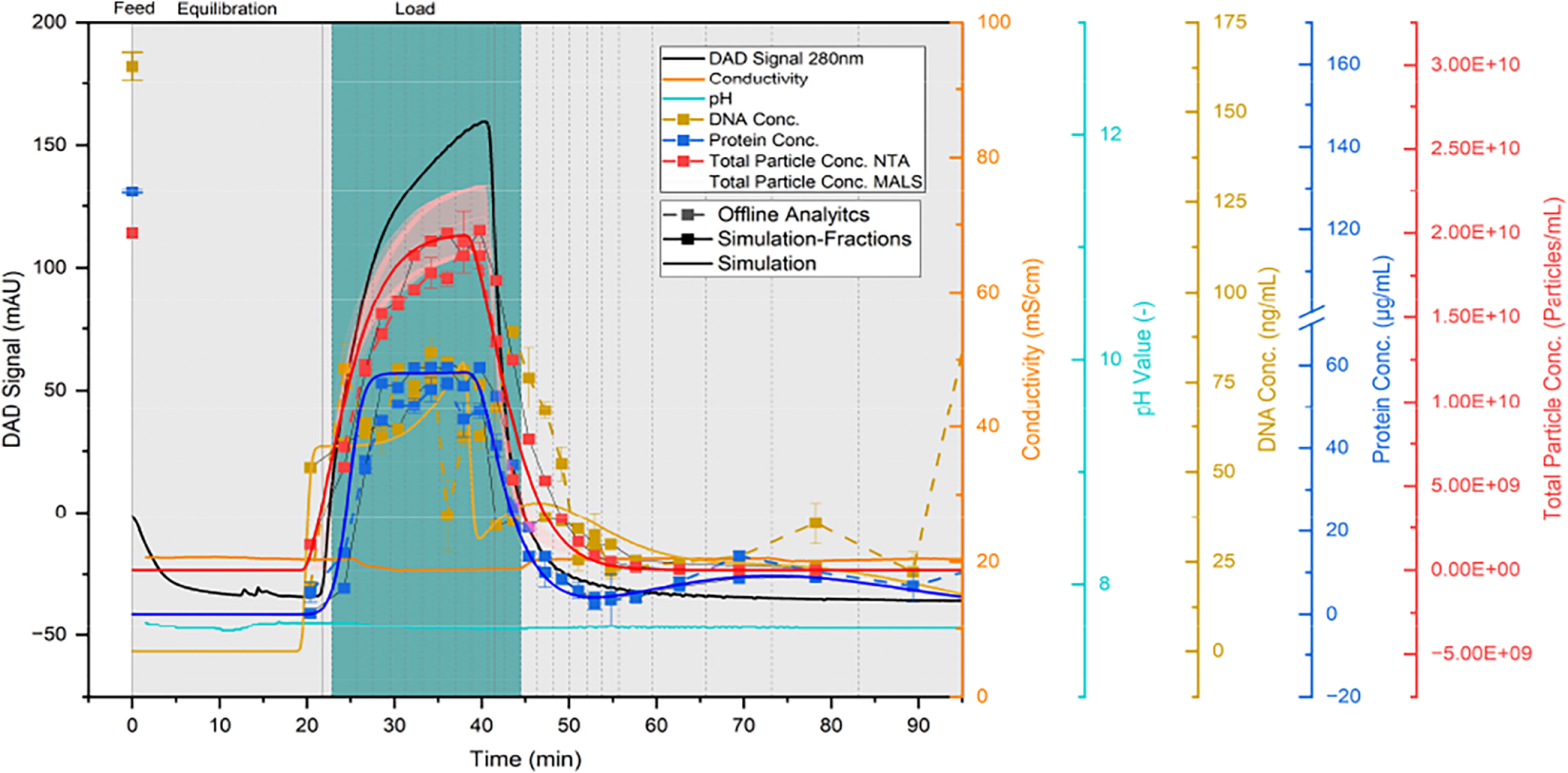
Chromatogram of multimodal SEC of the fraction analysis and the simulation of the elution profile and the respective fraction concentration.

**Figure 13:**
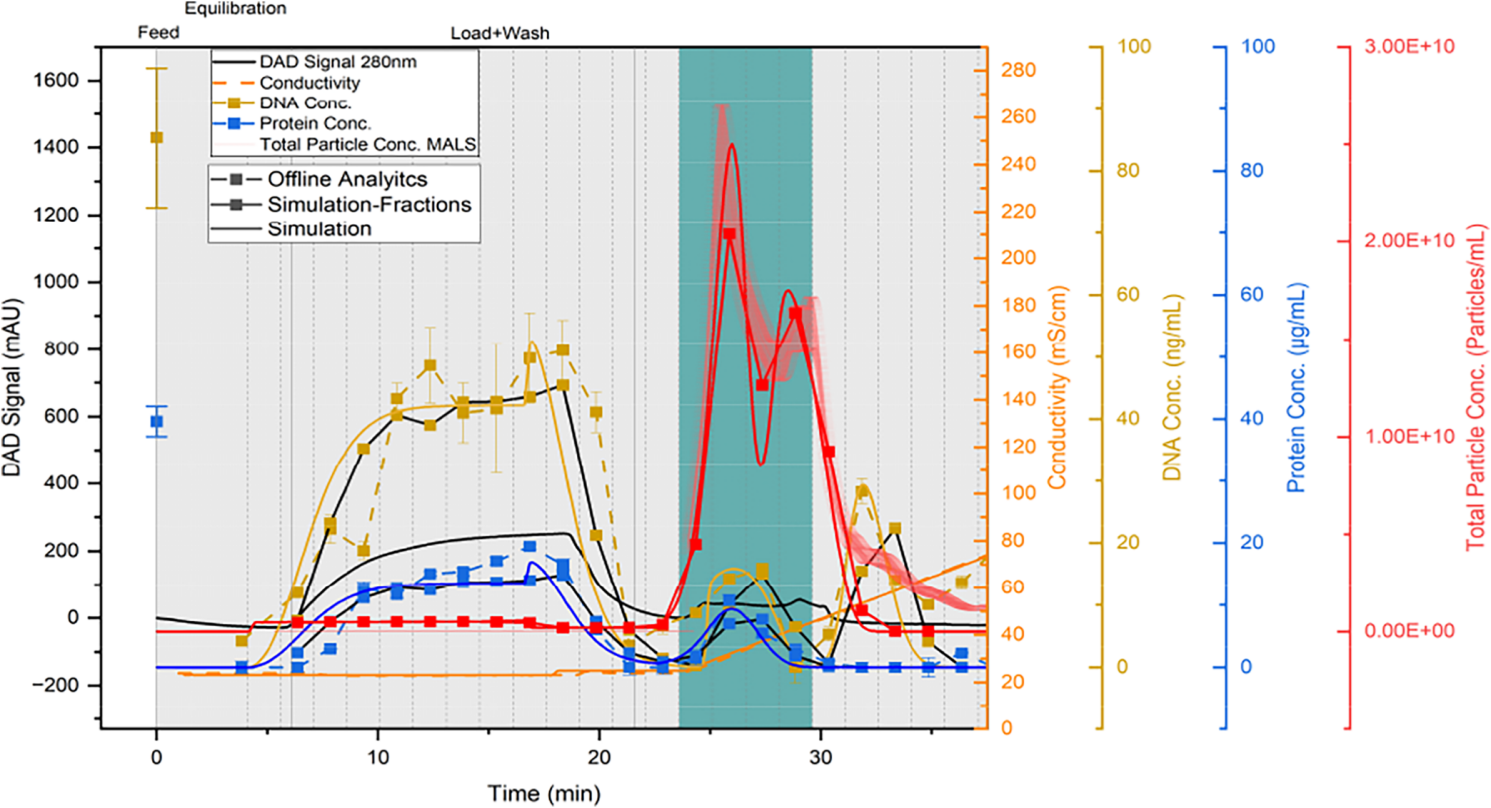
Chromatogram of the AEX chromatography of the fraction analysis and the simulation of the elution profile and the respective fraction concentration.

In the MM-SEC, the fluid dynamics parameters are determined via the CIP step at the end of the method for the modifier. The determined void fractions are the upper limit for determining the void fraction of the other larger components. The separation takes place in the isocratic range, so any dependence of the isotherm parameters on the modifier concentration can be neglected. A regression quality of *R* ^2^ = 0.89 for the exosomes, *R* ^2^ = 0.97 for the proteins, and *R* ^2^ = 0.62 for the DNA is achieved for the determination of the isotherm parameters.

By integration of a wash step and a gradient in the elution, the isotherm parameters in AEX are dependent on the modifier concentration. A regression quality of *R* ^2^ = 0.88 for exosomes, *R* ^2^ = 0.70 for DNA, and *R* ^2^ = 0.55 for proteins is achieved.

The aim of any optimization is to maximize productivity while keeping the purity requirements of the product within set limits. For MM-SEC, the process parameters that can be optimized are the volume of feed applied and the flow rate. These are shown in Figure 14. It is possible to increase the flow rate without reducing the purity of the product. Thus, an increase in productivity by a factor of around 2 can be achieved. However, an increase in feed loading also leads to an increase in impurities in the product pool and is therefore not recommended.

**Figure 14:**
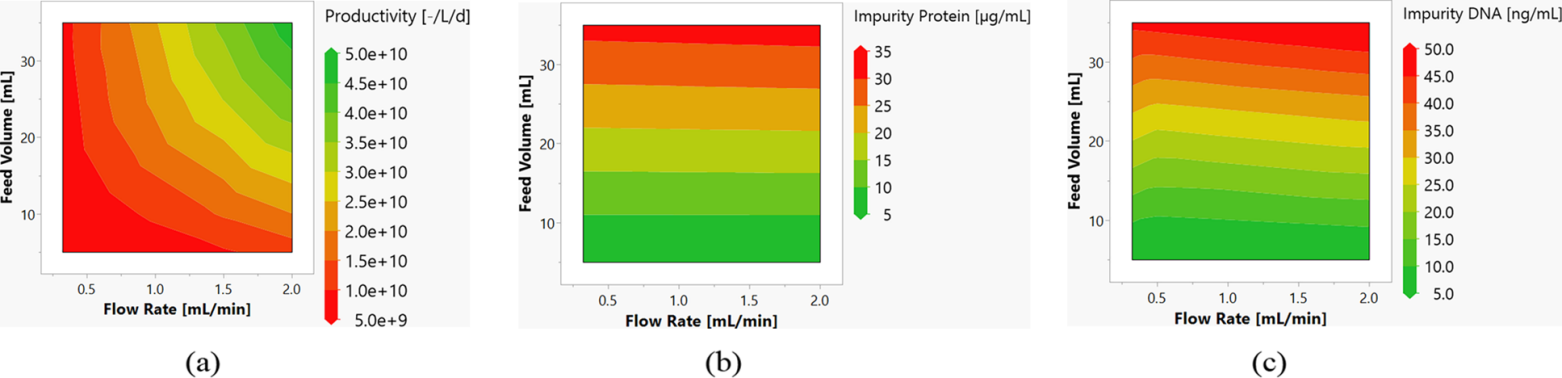
Contour plots of the productivity (a), the concentration of protein impurities (b), and DNA impurities (c) as a function of flow rate and feed volume.

With AEX, the process parameters gradient slope, feed volume, and concentration in the wash are examined. Increasing the feed volume in combination with a longer elution gradient also leads to a higher concentration of DNA in the pool. When the modifier concentration is increased in the wash step, it can be observed that exosomes elute there, which leads to a loss of yield and thus also to a reduction in productivity. Neither process parameter leads to any optimization of the process. The gradient slope can be significantly increased, whereby an improvement in productivity by a factor of 1.5 can be achieved without causing a deterioration in pool purity (Figure 15).

**Figure 15:**
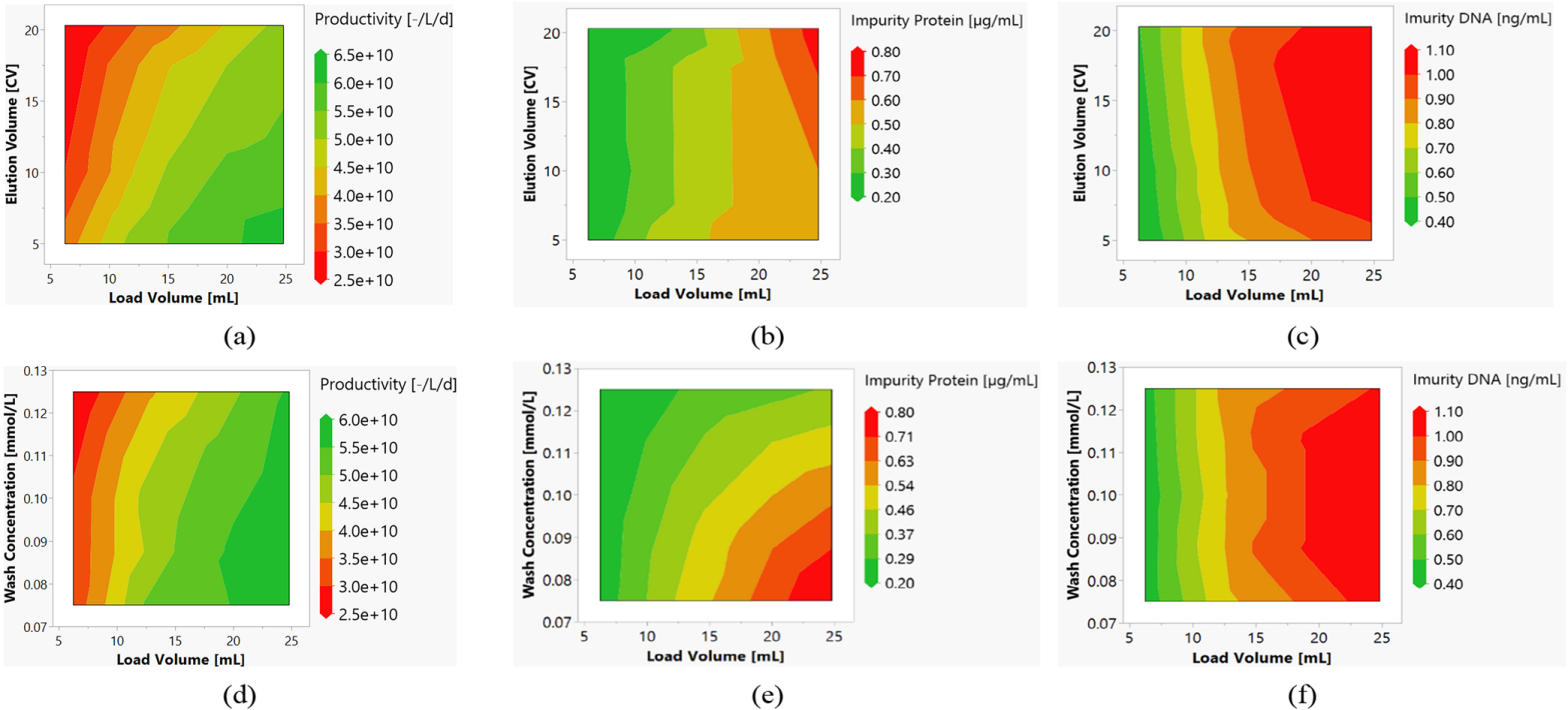
Contour plots of the productivity (a, d), the concentration of protein impurities (b, e), and DNA impurities (c, f) as a function of elution volume and feed volume (a–c) and wash concentration and feed volume (d–f).

## 5 Discussion and Conclusions

The process developed in this study comprises four purification steps. In the cultivation of adherently growing hMSCs, 0.89 ± 0.01 × 10^10^ EVs/mL were produced. Initial particulate contamination, such as dead or detached cells, was removed by depth filtration.

The harvest was then prepared for the subsequent chromatography steps using UFDF. In this process step, 70.8 ± 3.0% of the proteins and 77.1 ± 8.8% of the DNA could already be removed at an average LMH of 25.2 L/m^2^/h. This and the achieved recovery of 88.3 ± 4.6% are consistent with the observations of Zakhem et al.^67,125^

When loading the multimodal SEC with 1 CV of UFDF retentate, over 80% of the total protein could be removed, but only 44.3 ± 7.4% of the particles were recovered in the flow through and wash fractions. The remaining particles were found in the CIP step, with most particles eluting within the dead time and <3% of all particles were removed from the column with the CIP buffer. By increasing the loading volume to 3 CV, the recovery could be increased to >80%. At the same time, a comparable number of particles were found in the CIP step as with the load of 1 CV, which confirmed the previous assumption that particles are retained within the frit.

Since the purification using Capto Core 400 could not meet the regulatory threshold of 10 ng_DNA_/dose of dsDNA and 100 μg/mL of protein, an anion exchanger was investigated as an alternative. Processes described in the literature often use DNase to reduce the DNA concentration below the critical limit.^1,42,94,152,153^ The enzyme is usually added after harvesting, with an initial buffer exchange to the digestion buffer and following the digestion another buffer exchange to prepare for chromatography.^1,42,152^ In addition to longer process times, this also leads to higher requirements in purification and QA, as the added enzyme must be reliably removed in the downstream process.^149^

To investigate the necessity of adding DNase, the UFDF retentate was therefore loaded onto the AEX once without pretreatment and once after prior treatment with DNase. As the salt concentration in the chromatography buffer was presumably too high, it was not possible to achieve complete digestion of the DNA. However, effects on the elution profile of the DNA could be observed. Shorter DNA strands bind less well to the resin,^147^ which is why the DNA is primarily found in the flow through and at the beginning of the gradient. Without pretreatment, the DNA only elutes at conductivities >50 mS/cm, outside the elution of the product. Thus, a comparable DNA removal of approximately 95% is achieved. Although the regulatory requirements are met, the EV purity of 2.3–3.4 × 10^11^ Particles/mg_Protein_ is lower than in comparable processes.^1,67,126,152,155^

To take advantage of the high removal of proteins in the multimodal SEC and the high depletion of DNA in the AEX, the product fractions of the Capto Core 400 from the 3 CV load run were loaded onto the Poros HQ. This enabled an increase in EV purity by a factor of 50, reaching the upper end of documented purities.^1,67,126,152,155^ In addition, the amount of DNA could be reduced to 5.1 ng_DNA_/dose, which is comparable to literature processes (3.5–9.0 ng_DNA_/dose)^1,156,157^ and also enables a safer process that can better cope with possible process fluctuations. In addition, due to the low total protein concentration of 5.5 μg/mL, it can be assumed that the limit value of 100 μg/mL of HCP specified for biologics^12^ is also fulfilled.

The robust, scalable process results in an overall yield of 33.4%, in which the regulatory requirements are met, and 98.4% of the total protein and 99.3% of the DNA can be removed.

Validated process models and a reliable PAT strategy are essential for an automated process via APC in line with the QbD strategy. Using APC strategies, productivity increases of an additional 20% at 99.9% reliability have already been achieved for other entities such as nucleic acids, monoclonal antibodies, and VLPs.^103,109,110,121,123,166^ In addition, possible batch-to-batch variations can be compensated for by ensuring that the defined control spaces are kept according to QbD principles, as has been discussed for different ranges for the similar entity of lentivirus-like particles.^103^ In the case of uncompensatable variations, the digital twin approach can enable the early prediction of batch failures. The minimization of hold times resulting from the APC strategy has shown that time savings of a factor of 2 are possible.^110^ The applications of process models can start as a digital shadow before the transition toward full digital twin operation, but both implementations need a reliable QbD-derived control strategy and PAT to enable its execution. For successful implementation in real-time processes, appropriate interfaces must be created and integrated into a suitable, standardized software concept. Once this has been done, efficiency, innovation, and strategic growth can be achieved by companies that follow the Pharma 4.0 movement and thus the digital transformation.^167^

For this purpose, the required model parameters were determined from the experimental data, and the experimental results were successfully digitally reproduced. Together with the PAT solutions investigated, an APC concept was developed as shown in Figure 16. As the EVs are similar in size and filtration behavior, which is also underlined by the similar DSP, control and PAT are identical to the VLP process published earlier by Hengelbrock et al.^103^

**Figure 16:**
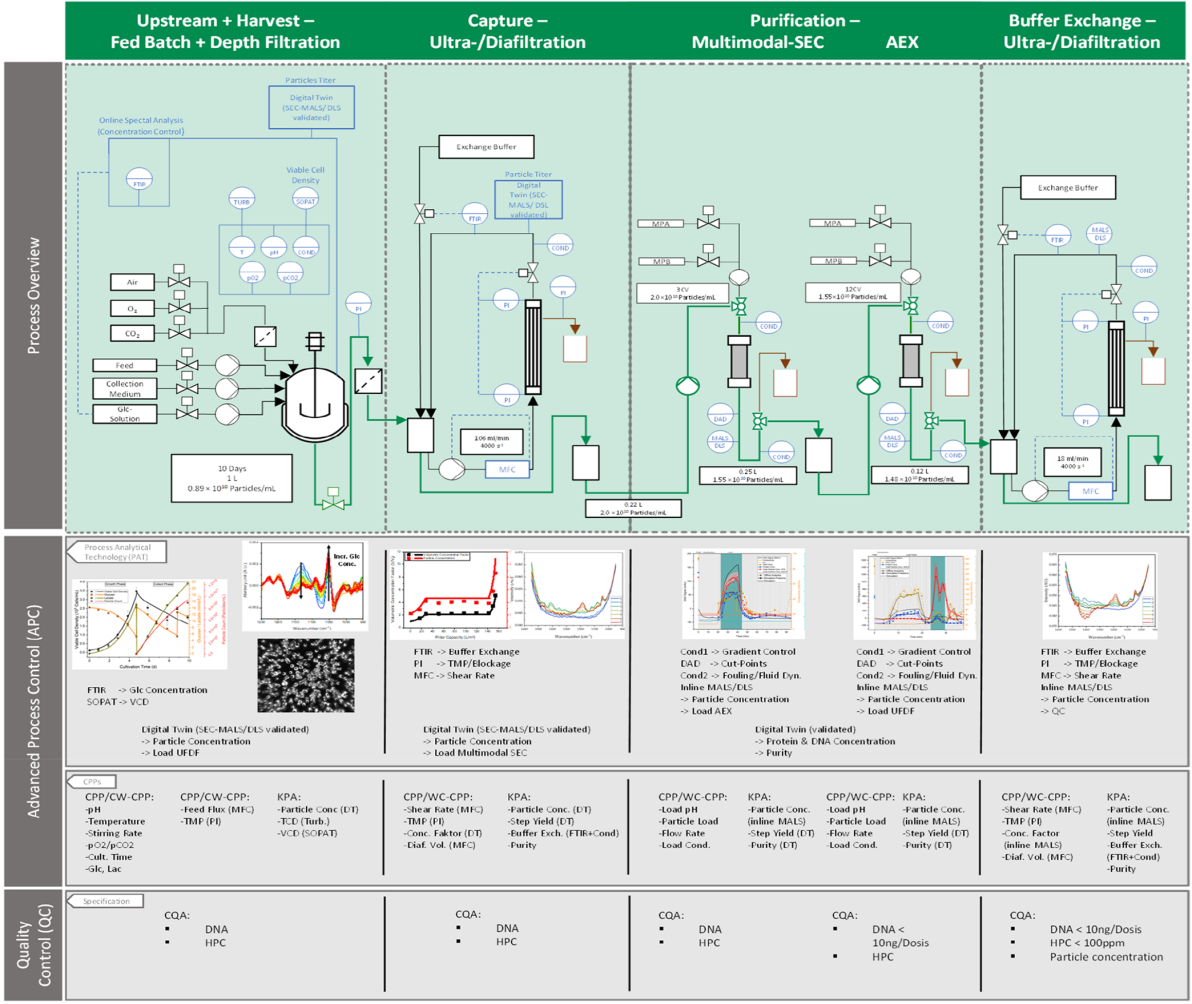
Process flowsheet with APC as a combination of DT and PAT.

In the USP, classic PAT such as O_2_, pH, and temperature sensors are used to monitor and control critical process parameters. In addition, it was shown that images taken with the help of the SOPAT Pa probe had the potential to track the VCD throughout the process. Only a simple neural network is required to process the characteristics of the images and the measurement settings. With further training and a more advanced neural network, it is possible to develop an even more precise and robust prediction of the VCD. In order to prevent substrate limitation, it is necessary to determine the glucose concentration. Raman and FTIR spectroscopy have been established for this purpose in various biological processes.^58,102,110,120^ In this study, it was shown that the spectrum of the collagen-coated microcarriers overlaps the media spectrum in the Raman. In addition, the intensity of the peaks is strongly dependent on the agitation rate. As the latter is not constant during the process, this leads to challenges in application as a PAT sensor. In contrast, these effects could not be observed in the FTIR spectra and a successful calibration and validation of the model for predicting the glucose concentration in the cultivation could be realized. Knowledge of the particle concentration is essential for successful cultivation, as well as for reducing holding times and preparing the next process steps accordingly. The process model calibrated and validated with offline particle concentrations can serve as a soft sensor.

A validated process calibrated to the specifics of the combination of product, impurities, and membrane enables accurate description of the filtration progress (see Figure 11). The application of such DT is demonstrated elsewhere numerous times, including end point prediction, unusual fouling mechanism identification, and optimized scheduling.^109,123^

The existing models for the VLP process^103,110^ were able to successfully describe the chromatographic process steps with adaptations for the multimodal SEC and adjustments of the model parameters to the EV process. Furthermore, the application of in-line MALS was demonstrated to determine the total number of particles. Comparable recoveries were measured as with NTA and ELISA, whereby MALS has advantages over NTA for unstable samples due to the possibility of real-time measurement. This allows holding times to be reduced and the EVs to be transferred to a stabilizing environment as quickly as possible via a final TFF step.

In summary, this study developed a robust, scalable process that meets the regulatory requirements with a dsDNA content of <10 ng_DNA_/dose and a total protein concentration of <100 μg/mL. With the developed predictive process models, the selection of suitable state-of-the-art PAT tools, and the proposal of an APC concept, the basis for an autonomous fully automated process in the sense of the QbD strategy for the production of cell and gene therapeutics was established.
